# Bayesian Learning of Distance Metrics Beyond RMSD for Biomolecule Alignment, Clustering, and Domain Identification

**DOI:** 10.1021/acs.jctc.6c01395

**Published:** 2026-10-01

**Authors:** Saumyak Mukherjee, Gerhard Hummer

**Affiliations:** † Department of Theoretical Biophysics, 28273 Max Planck Institute of Biophysics, 60438 Frankfurt am Main, Germany; ‡ Department of Chemical Sciences, Indian Institute of Science Education and Research, 760003 Berhampur, Odisha, India; § Institute of Biophysics, Goethe University Frankfurt, 60438 Frankfurt am Main, Germany

## Abstract

Root-mean-square deviation (RMSD) is the standard metric of structural comparison in molecular dynamics (MD) simulations. In its conventional form, RMSD assigns equal weight to all atoms regardless of mobility. Hence, flexible loops and disordered regions can dominate a global RMSD, while the rigid functional core contributes negligibly to the overall metric. To address this issue, we introduce the Bayes-optimal RMSD (*BRMSD*), which optimizes per-atom weights jointly with structural averages by maximizing a Bayesian posterior. In a trade-off between low RMSD and weight uniformity, a position-fluctuation parameter σ controls the transition from classical RMSD (σ → ∞) to a progressive focus on a rigid core (σ → 0). The BRMSD framework supports analysis modules for structural alignment, focused alignment onto a user-specified domain, trajectory smoothing, soft *K*-means conformational clustering, and rigid-domain identification. These modules are implemented in the open-source Python package BRMSD and benchmarked on two MD systems, the endoplasmic reticulum translocon-associated protein SND3 and the phosphotransferase adenylate kinase.

## Graphical abstract

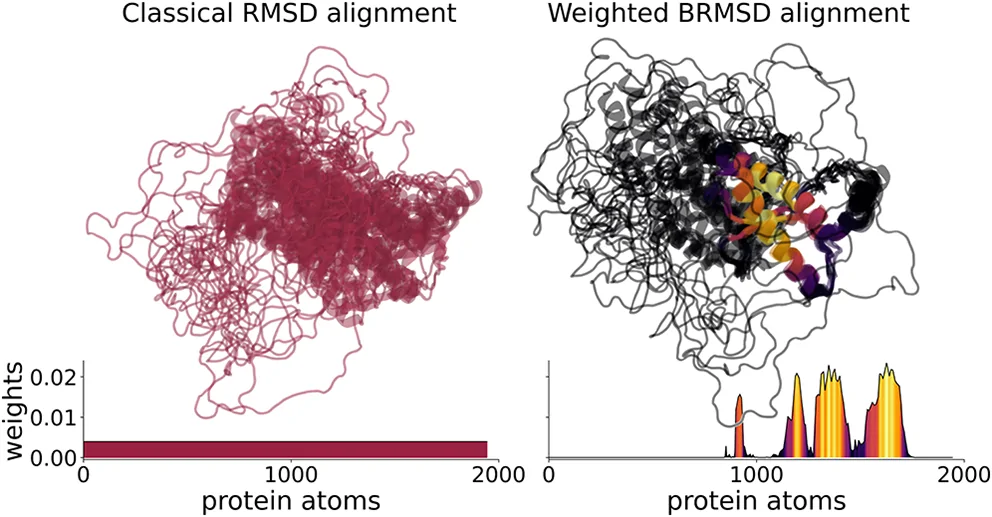

## 1 Introduction

Root-mean-square deviation (RMSD) is a fundamental metric, universally used to quantify structural similarity in MD simulations.^1–5^ Its utility comes from interpretability. It has units of length and a well-defined physical meaning as a measure of distance in conformation space. RMSD underlies convergence assessment, free-energy landscapes, conformational-state identification, and domain-motion analysis, all of which rely on the spatial deviation between conformations. The standard definition of RMSD assigns equal weight to a pre-selected set of atoms in a system, say the C_α_ atoms of a protein backbone. However, this a priori choice may conflate structurally informative and uninformative atoms. For example, in proteins with flexible loops, disordered termini, or mobile linkers, a handful of highly mobile residues can dominate the RMSD while focusing marginally on the conformational state of the functional core. Workarounds involve manual atom selection or iterative exclusion of outliers.^6^ However, it is difficult to generalize these approaches since they are user-dependent and require prior structural knowledge. This compromises reproducibility as MD simulation campaigns become increasingly automated to cover a wide range of molecular systems and states.

Rather than relying on ad hoc selection rules, a cleaner approach is to treat atomic weights as active variables within structural alignment optimization. Early protocols implemented static assignments, such as McLachlan’s mass-weighted superposition,^7^ which remain blind to the fluctuations of a given molecular dynamics trajectory. Later ensemble-based methodologies introduced by Damm and Carlson^8^ (via Gaussian variance scaling) and Theobald and Wuttke^9^ (via maximum-likelihood inference) succeeded in capturing trajectory-specific dynamics. However, these methods treat weight calculation and coordinate superposition as decoupled, sequential operations, leaving open the question of mathematical self-consistency between the weights and the resulting average structure.

Here, we bridge this gap by consolidating weight optimization and structural alignment into a single free-energy functional. In a Bayesian formulation, we derive the free energy as the negative logarithm of the Bayes posterior for the atom weights and reference structure. Minimizing the free energy then corresponds to a multi-objective optimization process that balances the competing demands to align the structures onto a quasi-rigid core and to include as many atoms into this core as possible. We refer to the RMSD calculated with optimized weights as “Bayes-optimal RMSD” (BRMSD). Equivalently, this trade-off between low mean-squared deviation (MSD) and near-uniform weights is achieved by combining the usual metric of MSD with a relative entropy regularizer that penalizes nonuniform atom weight distributions.^10,11^ Analogous approaches have been applied in reweighting and modeling of MD ensembles.^11–17^

We minimize a free-energy functional *G* to achieve low MSD while simultaneously penalizing deviations from the normalized prior weights *W*_α_ ≥ 0 with ∑_α_ *W*_α_ = 1. Here, we assume uniform prior weights, *W*_α_ = 1/*N*, where *N* is the total number of atoms. As penalty, we add the Kullback–Leibler (KL) divergence^18^ between the optimized atom weights ***w*** = {*w*_α_} and the prior ***W*** = {*W*_α_},*S*_KL_(***w*** ∥ ***W***) = ∑_α_ *w*_α_ ln­(*w*_α_/*W*_α_), to the total MSD from a reference structure. Simultaneous minimization over structure, rotations, and weights yields a fixed-point iteration that converges to the entropy-optimal weights.

The weight functional suppresses atoms in proportion to their mean-square fluctuations about the average structure, concentrating weight on rigid atoms and down-weighting flexible ones without the requirement of manual selection. The same functional form, adapted to incorporate window weights, domain bias, cluster assignments, or inter-domain overlap penalties, can be adapted to multiple distinct tasks, including structural alignment, focused alignment, trajectory smoothing, soft *K*-means clustering with cluster-specific atom weights, and rigid domain detection. All five modules share a single position-fluctuation parameter σ in units of Å. Large values of σ keep the KL divergence dominant, holding weights near the uniform prior and recovering classical RMSD. Smaller values of σ allow the concentration of weight onto the most-rigid atoms.

We benchmarked BRMSD on two systems chosen to represent complementary structural regimes. Alignment and smoothing analysis were performed on a 300 ns all-atom MD trajectory of SND3,^19,20^ an eukaryotic endoplasmic reticulum translocon-associated membrane protein, featuring a distinct transmembrane groove and flexible cytosolic claw domains. Clustering and rigid domain identification analyses were performed on the adenylate kinase (ADK) protein, which contains three distinct domains and has been shown to transition between open and closed states.^21^ For this analysis we use the directed transition trajectories distributed through the MDAnalysisData repository.^22–24^

## 2 Theory

### 2.1 Weighted Mean-Square Deviation

Let *x*_*i*_ = (*r*_*i*1_,*r*_*i*2_,···,*r*_*iN*_) denote the 3*N*-dimensional vector containing the three-dimensional Cartesian coordinates *r*_*i*α_ of the *N* atoms in molecular structure *i*. Given a normalized atom weight vector ***w*** = { *w*_α_} (α = 1,···, *N*) with ∑_α_ *w*_α_ = 1 and *w*_α_ ≥ 0, the weighted mean-square deviation (MSD) between two structures after optimal rigid-body superposition is given by

1 $$\mathrm{MSD}\left(x_{1},x_{2};w\right)=\min_{R}\sum_{\alpha }w_{\alpha }|r_{1\alpha }-Rr_{2\alpha }{|}^{2}$$

Here, *R* ∈ SE(3) denotes a rigid-body transformation. As a member of the special Euclidean group, *R* consists of a translation (of the entire structure) and a rotation (about its center, as defined by the weighted average of atom positions with weights *w*_α_). |·| represents the 3D Euclidean distance. The optimal *R* for a given weight vector ***w*** is found by the Kabsch algorithm.^2,3^ The root-mean-square deviation is RMSD = MSD^1/2^. Classical RMSD corresponds to the uniform prior *w*_α_ = *W*_α_ ≡ 1/*N* for all α.

### 2.2 Bayesian Formulation of RMSD Structural Alignment

In a Bayesian formulation of RMSD structural alignment, we assume a priori that configurations *x* in 3*N*-dimensional configuration space are distributed as

2 $$p_{0}\left(x|w,s,\sigma \right)\propto \exp\left(-\frac{\mathrm{MSD}\left(x,s;w\right)}{2\sigma^{2}}\right)$$

where *s* is the reference structure and σ is the expectation of the root-mean-squared fluctuation per atom. As a Bayesian prior for the weight vector, we assume that the optimal atom weight vector ***w*** entering the MSD is distributed as

3 $$q_{0}\left(w|W,\theta \right)\propto \exp\left(-\theta S_{\mathrm{KL}}\left(w∥W\right)\right)$$

where ***W*** is a vector of reference weights and θ is a confidence parameter. Analogous to Bayesian ensemble refinement,^12^ we then use the Bayes relation to define a Bayes posterior for *s* and ***w*** given a set of *M* configurations *x*_1_,*x*_2_,···,*x*_*M*_

4 $$\mathrm{posterior}\left(s,w|x_{1},x_{2},...;W,\theta ,\sigma \right)\propto \prod_{i}p_{0}\left(x_{i}|w,s,\sigma \right)q_{0}\left(w|W,\theta \right)=\exp\left(-g\right)$$

with an effective free energy

5 $$g=\sum_{i}\frac{\mathrm{MSD}\left(x_{i},s;w\right)}{2\sigma^{2}}+\theta S_{\mathrm{KL}}\left(w∥W\right)$$

The optimal reference and weight vector are then defined by maximizing the Bayes posterior, or equivalently by minimizing the effective free energy

6 $$\left(s,w\right)={\mathrm{argmax}}_{s,w}\mathrm{posterior}\left(s,w\right)={\mathrm{argmin}}_{s,w}g$$

Maximizing the posterior is unchanged if we multiply *g* by the constant 2σ^2^. Setting the prior confidence to θ = *M*/2 fixes the entropy penalty at a per-frame weight σ^2^ and a total weight of *M*σ^2^. With this choice, the confidence parameter is no longer free, and we minimize the effective free energy

7 $$G=\sum_{i}\mathrm{MSD}\left(x_{i},s;w\right)+M\sigma^{2}\sum_{\alpha }w_{\alpha }\ln\frac{w_{\alpha }}{W_{\alpha }}$$

with respect to *s* and ***w***. In the following, we use σ or

8 $$\theta =M\sigma^{2}$$

as fully equivalent parameters to control the trade-off between minimizing the MSD and the KL divergence. From here on θ denotes *M*σ^2^, not the prior confidence of eq 3. With this Bayesian perspective in mind, we refer to the RMSD alignment procedure using the optimal reference structure *s* and optimal weights ***w*** as “Bayes-optimal RMSD” (BRMSD).

We note the close analogy to an expression derived originally in the “ensemble refinement of SAXS” (EROS) method^11^ and then worked out in detail in ref 12. Here, the MSD term replaces the *χ* ^2^ error of ensemble refinement. As in ensemble refinement, an expression identical to eq 7 is obtained by combining the usual metric of MSD with a relative entropy regularizer to penalize nonuniform atom weight distributions.^10–12^

### 2.3 Structural Alignment

To find the weighted average structure *s* of an ensemble of *M* structures { *x*_*i*_}, we minimize *G* over the reference structure *s*, the rigid-body superpositions {*R*_*i*_} of *x*_*i*_ and *s*, and the atom weights ***w***, subject to the normalization condition of the weights. The first term in *G*, as given in eq 7, is the aggregate mean-square deviation from the average structure. The second term represents the relative entropy of ***w*** with respect to the uniform prior *W*_α_ = 1/*N*, scaled by θ = *M*σ^2^.

The minimization is achieved by setting derivatives of *G* with respect to *s*_α_ and *w*_α_ to zero. This yields the self-consistency equations

9 $$w_{\alpha }^{\left(n+1\right)}\propto W_{\alpha }\exp\left(-\frac{1}{M\sigma^{2}}\sum_{i}|s_{\alpha }^{\left(n\right)}-R_{i}^{\left(n\right)}r_{i\alpha }{|}^{2}\right)$$

10 $$s_{\alpha }^{\left(n+1\right)}=\frac{1}{M}\sum_{i=1}^{M}R_{i}^{\left(n+1\right)}r_{i\alpha }$$

where *R*_*i*_^(*n*)^ is the optimal rigid-body translation and rotation of *x*_*i*_ for weights ***w*** ^(*n*)^ with *s* ^*n*^ as reference, *R*_*i*_^(*n*)^ *r*_*i*α_ its application to atom α of *x*_*i*_, and the weights *w*_α_^(*n*)^ are normalized after each iteration step *n*. Starting from *s* ^(1)^ = *x*_1_ and *w*_α_^(1)^ = *W*_α_, eqs 10 and 9 are iterated toward convergence.

*G* has a lower bound of zero since the MSD term is a sum of squared norms and *S*_KL_(***w*** ∥ ***W***) ≥ 0 by Gibbs’ inequality.^10^ Each alternating minimization step decreases *G* monotonically. The weight update minimizes *G* exactly at fixed *s*, and the structure update minimizes the MSD term at fixed ***w***. Such a decrease of a function bounded from below guarantees convergence to a stationary point. Since *G* is non-convex, this point may be a local, rather than a global minimum. Therefore, although for alignment and smoothing, a single initialization from *s* ^(1)^ = *x*_1_ and *w*_α_^(1)^ = *W*_α_ may suffice, for clustering and domain identification, we run *n*_*r*_ random restarts and retain the solution with the lowest *G*.

The sum in the exponent of eq 9 is the per-atom mean-square fluctuation (MSF) about the current average. Accordingly, atoms with large fluctuations receive exponentially suppressed weights, while structurally rigid atoms are up-weighted. Atoms whose summed squared deviation ∑_*i*_ |*s*_α_ – *R*_*i*_ *r*_*i*_α|^2^ exceeds θ = *M*σ^2^ are exponentially suppressed, whereas those below retain near-uniform weight. Equivalently, an atom is suppressed once its mean-square fluctuation averaged over the *M* frames exceeds σ^2^. The weights are thus set by the per-atom mean-square fluctuation rather than by the number of frames *M* used to estimate it, provided the sampling is converged. At σ → ∞ the entropy term dominates and weights stay uniform, recovering classical RMSD. The optimal σ depends on the module and on the fluctuation amplitude of the system.

We define the Bayes-optimal RMSD (BRMSD) of frame *i* as the weighted deviation evaluated at the converged reference structure *s* and weights ***w***

11 $${\mathrm{BRMSD}}_{i}\equiv \mathrm{MSD}{\left(x_{i},s;w\right)}^{1/2}={\left[\sum_{\alpha }w_{\alpha }|s_{\alpha }-R_{i}r_{i\alpha }{|}^{2}\right]}^{1/2}$$

Values reported as BRMSD are the mean and standard deviation of BRMSD_*i*_ over all frames *i*. We measure how many atoms effectively carry the alignment by the inverse participation ratio of the weight distribution (eq 12)­

12 $$n_{\mathrm{eff}}={\left(\sum_{\alpha }w_{\alpha }^{2}\right)}^{-1}$$

where *n*_eff_ equals *N* for uniform weights and decreases as weight concentrates, reaching one when a single atom carries all of the weight.

### 2.4 Focused Alignment

The BRMSD alignment concentrates weight on the most globally rigid regions of the protein, such as the TMD groove in SND3. However, the most functionally relevant regions may not always coincide with the rigid structural core. For instance, a flexible domain critical for substrate binding will warrant non-negligible weight alongside the globally rigid scaffold. Hence, depending on the problem at hand, one may wish to focus the alignment on a given domain of interest *D* while still including the remainder of the structure. We augment the free-energy functional of eq 7 with a concentration penalty on atoms in *D* (eq 13).

13 $$G=\sum_{i}\mathrm{MSD}\left(x_{i},s;w\right)+\theta \sum_{\alpha }w_{\alpha }\ln\frac{w_{\alpha }}{W_{\alpha }}+\mu \sum_{\alpha \in D}w_{\alpha }\ln\left(n_{D}w_{\alpha }\right)$$

where *n*_*D*_ is the number of atoms in *D* and μ > 0 controls the strength of the concentration bias toward *D*. Taking the derivative with respect to *w*_α_ yields two separate update rules.

14 $$w_{\alpha }^{\left(n+1\right)}\propto W_{\alpha }\exp\left(-\frac{\sum_{i}|s_{\alpha }^{\left(n\right)}-R_{i}^{\left(n\right)}r_{i\alpha }{|}^{2}}{\theta }\right),\alpha \notin D$$

15 $$w_{\alpha }^{\left(n+1\right)}\propto \frac{W_{\alpha }^{\theta /\left(\theta +\mu \right)}}{n_{D}^{\mu /\left(\theta +\mu \right)}}\exp\left(-\frac{\sum_{i}|s_{\alpha }^{\left(n\right)}-R_{i}^{\left(n\right)}r_{i\alpha }{|}^{2}}{\theta +\mu }\right),\alpha \in D$$

Atoms outside *D* follow the standard alignment update (with equivalent eqs 14 and 9), whereas atoms inside *D* experience a reduced effective concentration parameter θ + μ and an additional prior bias *W*_α_^θ/(θ+μ)^ that amplifies their weight relative to atoms outside *D*. In the limit μ ≫ θ, the atoms in *D* receive weight *w*_α_ ∝ *n*_*D*_^–1^ (uniform within *D*) regardless of their MSF. As before, all weights are normalized after each update, and the structure update follows eq 10.

The dimensionless ratio μ/θ controls the degree of domain focusing. A practical criterion for selecting the operating point (μ/θ)_op_ is that *n*_eff_ should remain above |*D*|. If *n*_eff_ < |*D*|, the alignment is effectively performed on fewer atoms than the domain contains, which can cause numerical instability.

### 2.5 Nonuniform Priors

The prior weights *W*_α_ in eq 7 have so far been uniform, *W*_α_ = 1/*N*. Any normalized prior with *W*_α_ ≥ 0 and ∑_α_ *W*_α_ = 1 can be used in its place, and the fixed-point equations are unchanged. We consider two constructions. A *domain prior* places a fraction *c* of the prior mass on a chosen set *D* of *n*_*D*_ atoms,

16 $$W_{\alpha }=\frac{c}{n_{D}}\left(\alpha \in D\right),,W_{\alpha }=\frac{1-c}{N-n_{D}}\left(\alpha \notin D\right)$$

with *c* = *n*_*D*_/*N* recovering the uniform prior and 0 < *c* < 1. A *mass prior* sets *W*_α_ = *m*_α_/∑_β_ *m*_β_ with *m*_α_ the mass of atom α, which makes eq 7 a Bayesian counterpart of mass-weighted superposition.^7^

A domain prior is not a reparameterization of the focusing penalty of eq 13. Written in logarithmic form, the alignment update of eq 9 reads ln *w*_α_ = ln* W*_α_ −*d*_α_/θ + const, with *d*_α_ = ∑_*i*_ |*s*_α_ −*R*_*i*_ *r*_*i*_α|^2^, the summed squared deviation of atom α. A prior therefore enters as an additive constant. It raises every atom of *D* by the same amount ln­[*c*(*N*–*n*_*D*_)/((1–*c*) *n*_*D*_)] and leaves the spread of weights within *D* unchanged. The focusing penalty instead replaces θ by θ + μ for α ∈ *D* (eq 15), which compresses that spread. The two limits differ accordingly. As μ → ∞ the weights within *D* approach 1/*n*_*D*_ irrespective of atom mobility, whereas as *c* →1 the weight stays distributed within *D* in proportion to exp­(−*d*_α_/θ) and so concentrates on the most rigid atoms of *D*. The prior expresses which atoms are expected to matter, and μ sets how uniformly they are treated once chosen.

### 2.6 Trajectory Smoothing

Structural alignment averages uniformly over all *M* frames. High-frequency positional noise from thermal fluctuations is thereby retained in each individual frame, even after alignment. To suppress this noise while preserving slow conformational dynamics, we replace the global average with a local one centered on each frame *j*. We introduce frame-dependent weights *p*(*i*|*j*) ≥ 0, ∑_*i*_ *p*(*i*|*j*) = 1, that localize structure *i* relative to frame *j*. A triangular window of half-width *W* sets *p*(*i*|*j*)∝ max­(0, *W* – |*i* – *j*|) or a uniform window can be used. The smoothed-frame functional replaces eq 7 by

17 $$G_{j}=\sum_{i}p\left(i|j\right)\mathrm{MSD}\left(x_{i},s_{j};w_{j}\right)+\theta \sum_{\alpha }w_{j\alpha }\ln\frac{w_{j\alpha }}{W_{\alpha }}$$

minimized independently for each frame *j*. The fixed-point equations are identical in form to eqs 9 and 10 with the uniform sum replaced by the windowed sum ∑_*i*_ *p*(*i*|*j*). Because the window weights are normalized (∑_*i*_ *p*(*i*|*j*) = 1), this windowed sum is a per-frame average, with an entropy penalty of θ = σ^2^ (and an effective frame count of *M* = 1). The converged *s*_*j*_ is the smoothed coordinate of frame *j*, and the collection {*s*_*j*_} constitutes the smoothed trajectory.

### 2.7 Conformational Clustering

Structural alignment identifies a single average structure and a single weight vector for the full ensemble. When the ensemble spans multiple conformational states, a single average is insufficient. We extend the alignment framework to *K* cluster centers by replacing the uniform frame average with a soft assignment. Each frame *i* is assigned to cluster *a* with responsibility^25^ *q*(*a*|*i*)≥ 0, ∑_*a*_ *q*(*a*|*i*) = 1. Each cluster carries its own center *s*_*a*_ and, crucially, its own atom weight vector ***w***_*a*_. The alignment metric thus adapts to the internal rigidity pattern of each conformational state rather than imposing a single global metric. The joint free-energy functional is given by eq 18.

18 $$G=\sum_{a}\sum_{i}q\left(a|i\right)\sum_{\alpha }w_{a\alpha }\left|s_{a\alpha }-R_{\mathrm{ia}}r_{i\alpha }{|}^{2}+\theta \sum_{a}\sum_{\alpha }w_{a\alpha }\ln\frac{w_{a\alpha }}{W_{\alpha }}+\tau \sum_{i}\sum_{a}q(a\right|i)\ln\left(\mathrm{Mq}\left(a|i\right)\right)$$

Here, the third term penalizes deterministic assignments (large τ encourages soft, diffuse responsibilities, small τ recovers hard *K*-means). Setting ∂*G*/∂*q*(*a*|*i*) = 0 subject to ∑_*a*_ *q*(*a*|*i*) = 1 via a Lagrange multiplier yields the softmax responsibility update (eq 19), where *s*_*a*_ is the center of cluster *a*, θ controls atom weight concentration (eq 8), and τ > 0 (units Å^2^) controls cluster assignment softness. The fixed-point iteration is

19 $$q^{\left(n+1\right)}\left(a|i\right)\propto \exp\left(-\frac{\sum_{\alpha }w_{a\alpha }^{\left(n\right)}|s_{a\alpha }^{\left(n\right)}-R_{\mathrm{ia}}^{\left(n\right)}r_{i\alpha }{|}^{2}}{\tau }\right)$$

20 $$w_{a\alpha }^{\left(n+1\right)}\propto W_{\alpha }\exp\left(-\frac{\sum_{i}q^{\left(n+1\right)}\left(a|i\right)|s_{a\alpha }^{\left(n\right)}-R_{\mathrm{ia}}^{\left(n\right)}r_{i\alpha }{|}^{2}}{\theta }\right)$$

21 $$s_{a\alpha }^{\left(n+1\right)}=\frac{\sum_{i}q^{\left(n+1\right)}\left(a|i\right)R_{\mathrm{ia}}^{\left(n+1\right)}r_{i\alpha }}{\sum_{i}q^{\left(n+1\right)}\left(a|i\right)}$$

The cluster-center *s*_*a*α_ is the responsibility-weighted average structure and represents the fixed point that minimizes the weighted MSD across all frames. Since each cluster has its own weight vector ***w***_*a*_, the alignment metric adapts to each cluster’s internal rigidity pattern.

### 2.8 Rigid Domain Identification

Conformational clustering partitions frames into groups (clusters), whereas rigid-domain identification partitions atoms into groups (domains). Both share the BRMSD free energy structure, but the variable of interest shifts from frame assignments *q*(*a*|*i*) to atom weight vectors ***w***_*k*_.

The identification of rigid domains is done by sequential peeling of atoms. A series of independent optimizations with *K* = 1 domain is performed on shrinking atom pools. At each step, the BRMSD functional (eq 7) is minimized on the current pool to obtain a single weight vector ***w*** concentrated on the most rigid substructure within that pool. Atoms with *w*_α_ > *t*·max_β_ *w*_β_ are assigned to the current domain and removed from the pool. The threshold *t* controls pool composition for subsequent steps. Small values over-claim atoms, depleting later pools, and large values underclaim, leaving rigid residues in the pool that can dominate subsequent steps. The procedure repeats on the remaining atoms until no atoms satisfy the threshold or *K* domains have been recovered. The peeling order is data-determined, such that the most globally rigid substructure is identified first. Hence, domains emerge in order of decreasing rigidity. At each step, θ = *M*σ^2^ is held fixed and independent of the pool size, preserving the physical meaning of σ as the per-atom root-mean-squared fluctuation (RMSF) threshold across all steps. Domain detection quality is assessed by the Jaccard index *J*(*A*, *B*) = |*A* ∩*B*|/|*A* ∪*B*| between each recovered domain and the reference assignment.

An alternative formulation seeks *K* non-overlapping weight vectors simultaneously, enforcing mutual exclusivity through an overlap penalty on the dot products ***w***_*j*_· ***w***_*k*_.

22 $$G=\sum_{k}\sum_{i}\mathrm{MSD}\left(x_{i},s_{k};w_{k}\right)+\theta \sum_{k}\sum_{\alpha }w_{k\alpha }\ln\frac{w_{k\alpha }}{W_{\alpha }}+\frac{\mu }{2}\sum_{j<k}{\left(w_{j}\cdot w_{k}\right)}^{2}$$

The weight update becomes

23 $$w_{k\alpha }^{\left(n+1\right)}\propto W_{\alpha }\exp\left(-\frac{\sum_{i}|s_{k\alpha }^{\left(n\right)}-R_{\mathrm{ki}}^{\left(n\right)}r_{i\alpha }{|}^{2}}{\theta }-\mu \sum_{j\neq k}\left(w_{j}^{\left(n\right)}\cdot w_{k}^{\left(n\right)}\right)w_{j\alpha }^{\left(n\right)}\right)$$

This update is obtained by differentiating *G* with respect to *w*_*k*α_, adding a Lagrange multiplier for the simplex constraint ∑_α_ *w*_*kα*_ = 1, and setting the derivative to zero. The overlap gradient μ∑_*j≠k*_ (***w***_*j*_· ***w***_*k*_)*w*_*jα*_ enters additively in the exponent, coupling domain *k*’s weights to all other domains. Since the coupling is simultaneous, the weight updates for different *k* are not independent. In practice, simultaneous optimization can fail when the overlap-penalty gradient is too weak to prevent weight vectors from collapsing onto the globally most rigid region (see Section 3). Sequential peeling resolves this by construction.

Implementation details, including timing benchmarks for all modules (SI Table S1), are provided in the Supporting Information.

### 2.9 Extension to Mahalanobis-like RMSD Metric

For completeness, we also sketch an extension to a Mahalonobis-like RMSD that accounts for anisotropic fluctuations in atom positions. Let us assume that we have an initial alignment, which gives us the aligned structures *x*_*i*_. In a form of PCA,^26^ we then construct the average *s* = ∑_*i* = 1_^M^ *x*_*i*_/*M* of the *M* structures and the covariance matrix *C* = ∑_*i* = 1_^*M*^δ *x*_*i*_δ *x*_*i*_^*T*^/*M* with δ*x*_*i*_ = *x*_*i*_–*s* and *T* the transpose. A spectral decomposition of *C* (or a singular value decomposition of the *x*_*i*_) then gives us *n* eigenvalues λ_*i*_ > 0 with orthonormal eigenvectors *v*_*i*_, i.e., *v*_*i*_^*T*^v_*j*_ = δ*_ij_* with δ_*ij*_ the Kronecker delta. At least six of the eigenvalues will be zero, corresponding to rigid-body translations and rotations, *n* ≤ 3*N*–6. We define the free energy by giving weights *w*_*j*_ to the PCA modes *v*_*j*_ as

24 $$G=\sum_{i=1}^{M}\sum_{j=1}^{n}w_{j}{\left[v_{j}^{T}\left(Rx_{i}-s\right)\right]}^{2}+\theta \sum_{j=1}^{n}w_{j}\ln\frac{w_{j}}{W_{j}}$$

where ***R*** = *I*_*N*_⊗*R* depends on *i* and applies the translation and rotation atom-wise to the configurations *x*_*i*_. Minimization with respect to *s* gives the average position, *s* = ∑_*i* = 1_^*M*^ ***R*** *x*_*i*_/*M*, as before. Setting the derivative with respect to the normalized weights *w*_*j*_ to zero gives us an update rule for the mode weights

25 $$w_{j}\propto W_{j}e^{-\sum_{i=1}^{M}{\left[v_{j}^{T}\left(Rx_{i}-s\right)\right]}^{2}/\theta }$$

However, we have not currently implemented this procedure because the rigid-body alignment code to optimize *R* will have to be adapted to the more general metric. After iteration to self-consistency, one would then interpolate between an alignment according to the Mahalanobis distance (θ = 0) and the usual Euclidean distance (θ → ∞).

### 2.10 Parameter Classification

The reference conformation(s) and atom weights, *s* and ***w*** (or, *s*_*a*_, ***w***_*a*_, and *q*(*a*|*i*) for clustering and *s*_*k*_, ***w***_*k*_ for domain identification) are inferred by minimizing *G*. The following user-specified hyperparameters are fixed before each optimization, and possibly tuned according to the diagnostics described Section 3: σ (equivalently θ = *M*σ^2^), the prior weight vector ***W*** together with any parameter that defines it (the mass ***W*** ∝ ***m*** of Section 2.5, or the domain-prior mass *c* of eq 16), and the module-specific parameters, such as τ and *K* for clustering, μ/θ for focused alignment, μ for the domain-identification overlap penalty (simultaneous mode), the peeling threshold *t*, the smoothing window half-width *W* and its shape (triangular or uniform), and the number of random restarts *n*_*r*_ used for clustering and domain identification. *M* (the number of frames) and *N* (the number of atoms in the selection) are problem-specific quantities set by the input trajectory and atom selection.

## 3 Results and Discussion

### 3.1 Systems

We benchmarked BRMSD on two systems that represent complementary challenges. For alignment and smoothing, we used SND3, which is a 191-residue eukaryotic translocon-associated protein recently resolved by cryo-EM at 3.1 Å resolution (PDB: 9I78).^19^ Three transmembrane helices (TMD-2, residues 28 to 60; TMD-3, residues 86 to 115; TMD-4, residues 119 to 134) form a buried TM groove that constitutes the rigid functional core. An additional, shorter transmembrane helix (TMD-1, residues 5 to 21) is embedded in the membrane and is less rigid than the other three TMDs (it serves as the target domain for the focused alignment benchmark). Two cytosolic claw domains (N-claw, residues 61 to 85; C-claw, residues 136 to 173) and a C-terminal tail (residues 174 to 191) are substantially more mobile. We analyzed a 300 ns all-atom MD trajectory of the isolated protein chain, sampled at 100 ps intervals. SND3 is one subunit of a larger translocon assembly, and the same simulation contains the full six-chain complex (SEC61α, SEC61β, SEC61γ, CCDC47, SND3, and TRAPα; 19,811 atoms). We use that complex separately to test the method on a multi-subunit system. The SND3 trajectory samples equilibrium fluctuations around a single mean structure without discrete conformational states.

The conformational clustering and domain identification were benchmarked on adenylate kinase (ADK), a phosphotransferase that undergoes a large-scale open-to-closed conformational transition upon substrate binding.^21^ Crystallography and mutational studies have established three structurally distinct regions in ADK to single-residue precision, namely, the CORE (residues 1 to 29, 60 to 121, and 160 to 214), NMPbind (residues 30 to 59), and LID (residues 122 to 159) domains.^21^ The two endpoint crystal structures, open (PDB: 4AKE) and closed (PDB ID: 1AKE), are characterized by NMPbind-LID distances of 32.1 and 19.6 Å, and LID-CORE distances of 40.0 and 30.0 Å, respectively. To evaluate clustering performance, we utilized 200 independent Dynamic Importance Sampling (DIMS) trajectories^27^ (with a total of 19,691 frames) that span this open-to-closed transition. This ensemble represents a non-equilibrium transition rather than a continuous equilibrium trajectory. For domain detection validation, we employed 28,282 Framework Rigidity Optimized Dynamics Algorithm (FRODA) frames.^28^ FRODA propagates rigid-body constraints on covalent geometry. Intra-domain RMSF is thus substantially lower than inter-domain RMSF by construction.

### 3.2 Alignment

We scanned σ from 0.1 to 10 Å on the SND3 trajectory (Figure 1). As σ decreases, the per-residue weights *w*_residue_ grow monotonically in the TM groove (TMD-2, TMD-3, TMD-4) while collapsing toward zero in the more mobile regions (Figure 1A). The per-residue RMSF profile is mostly insensitive to σ (Figure 1B), confirming that the weight distribution robustly identifies the same rigid core regardless of regularization strength.

**Figure 1:**
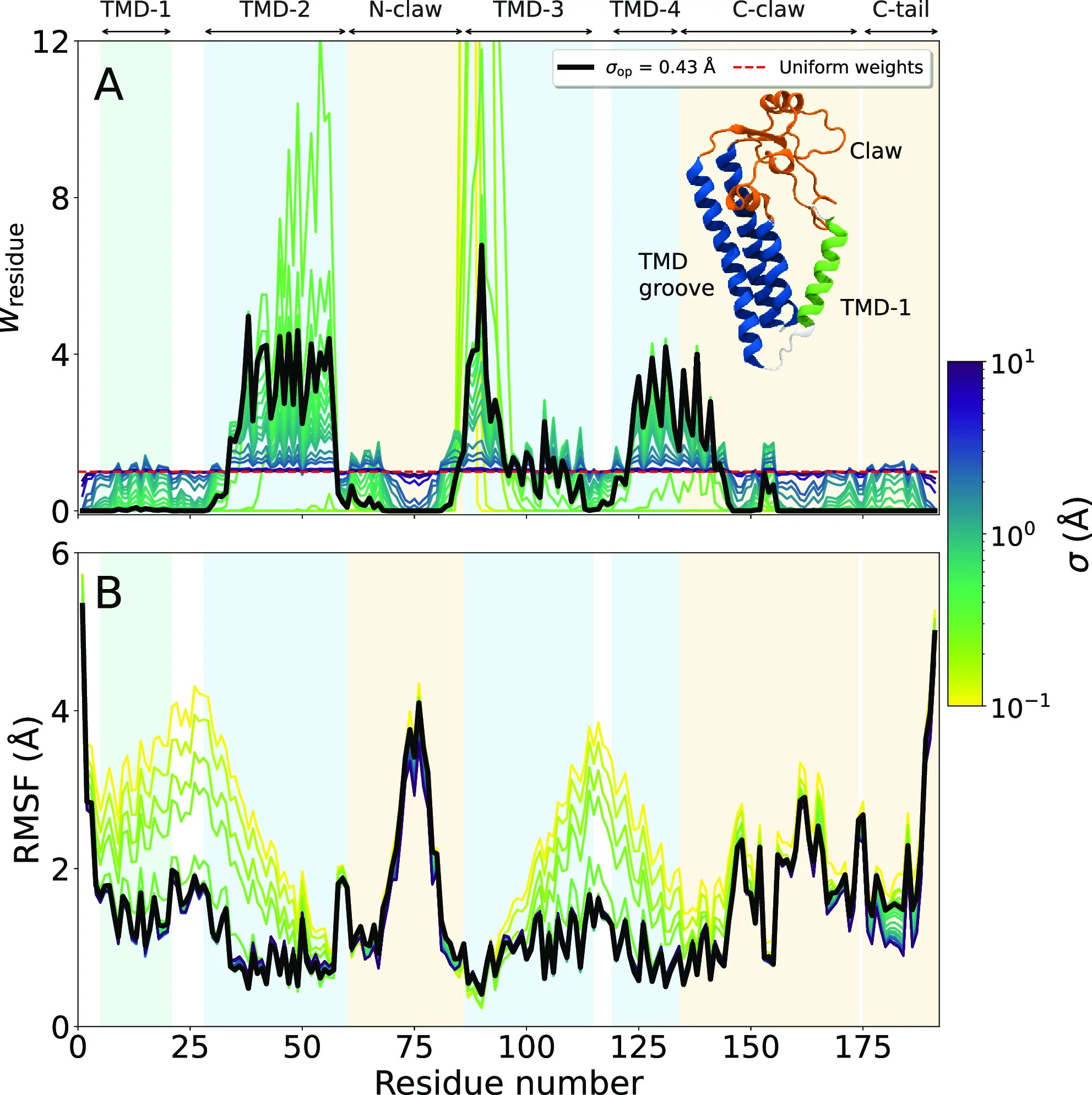
σ sweep for SND3 structural alignment is shown in two panels. (A) Per-residue weight *w*_residue_ (mean atom weight of a residue, scaled so a uniform weight equals one) as a function of residue number for σ = 0.1 to 10.0 Å, colored by σ (colorbar). The converged solution at the selected operating point σ_op_ = 0.43 Å is overlaid as a thick black curve in both panels. Domain shading indicates TMD-1 (green), TMD-2/TMD-3/TMD-4 (blue, TM groove), and N-claw/C-claw/C-tail (orange). The SND3 structure (PDB: 9I78), colored according to the domains, is shown as an inset. As σ decreases, weight concentrates progressively in the TM groove. (B) Per-residue RMSF for the same σ values. The profile is independent of σ (other than at very low σ, due to over-regularization), confirming that the weight redistribution does not alter the underlying fluctuations.

The role of σ is not to alter which residues are mobile, but to control the sharpness of the core-periphery boundary used for alignment and domain decomposition. Larger σ yields a diffuse weighting that spreads weight across many residues, while smaller σ sharpens the boundary, concentrating weight on the most rigid atoms. However, excessively small σ under-regularizes the distribution, collapsing the effective alignment set to too few atoms and risking sensitivity to local structural noise rather than global domain motion.

The optimal *σ* is chosen from two diagnostics plotted in Figure 2A, namely the ratio *w*_groove_/*w*_rest_ with *w*_D_ = ∑_α∈D_ *w*_α_ (*D* = groove, rest) (shown in red) and the effective atom count *n*_eff_ (eq 12) (shown in green). We define σ_op_ as the smallest σ at which *n*_eff_ ≥ 0.20 *N*. At this value, at least one-fifth of all atoms contribute to the alignment and weight concentration on the rigid core is near its practical maximum. At σ_op_ = 0.43 Å, the ratio reaches 6.5 and *n*_eff_ = 604. The groove residues thus carry the dominant weight while a statistically meaningful fraction of the chain (604 of 3013 atoms) still contributes to the alignment. The cumulative weight-MSF Lorenz curve at σ_op_ quantifies this discrimination. The 50% most-rigid atoms carry approximately 90% of the total weight (SI Figure S1).

**Figure 2:**
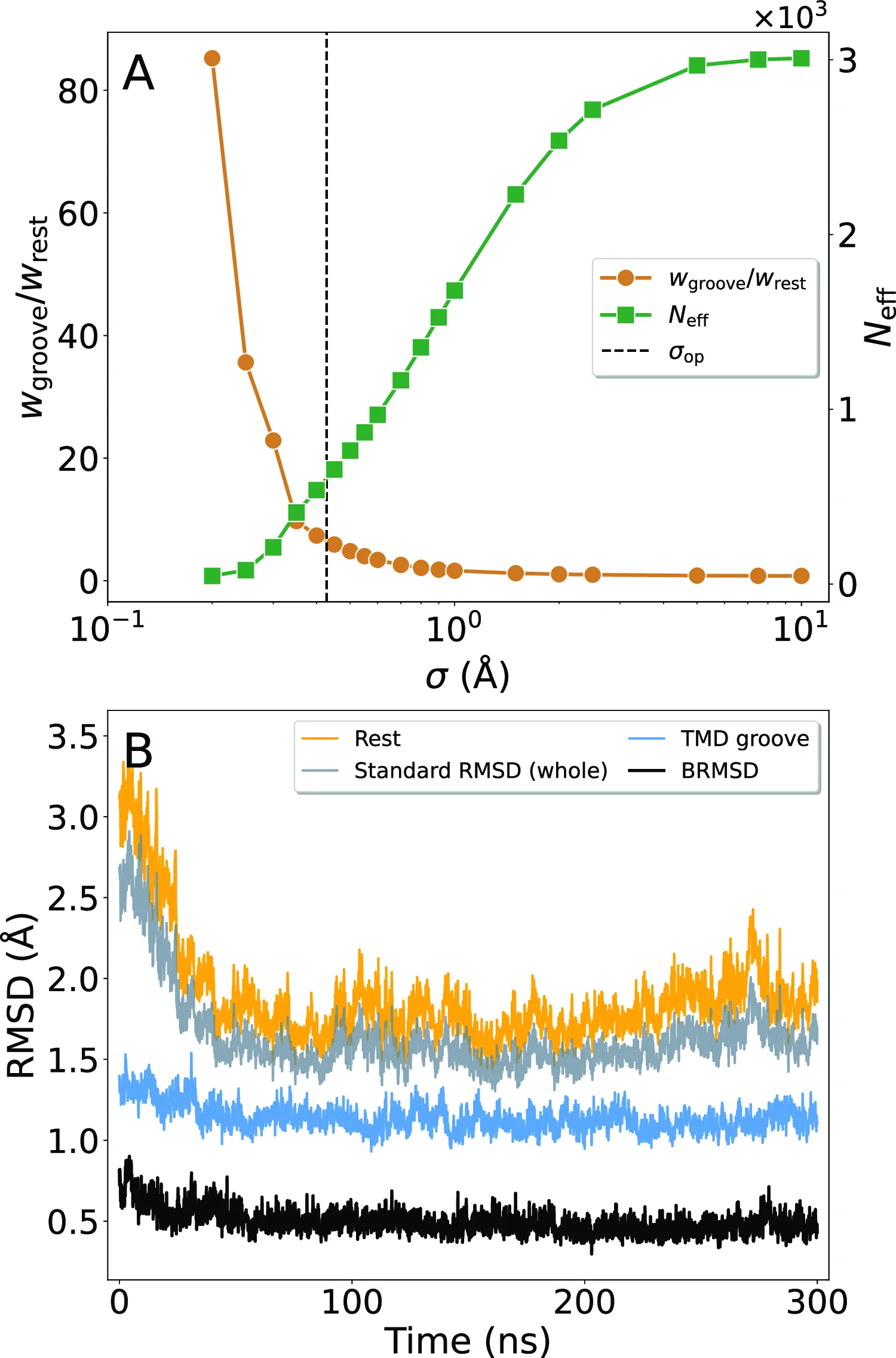
Optimal σ selection and alignment quality are shown in two panels. (A) Weight ratio *w*_groove_/*w*_rest_ (red, left axis) and effective atom count *n*_eff_ (green, right axis) are plotted versus σ (log scale). At σ_op_ = 0.43 Å (dashed line), the ratio is 6.5 and *n*_eff_ = 604. (B) RMSD time series over the 300 ns trajectory for the TM groove (blue), the non-groove region (orange), the overall standard RMSD (gray), and the Bayes-optimal RMSD at σ_op_ (black, eq 11). The BRMSD (0.50 ± 0.08 Å) lies below the unweighted measures, including the groove RMSD, and is ∼3 times smaller and ∼10 times less variable than the standard all-atom RMSD (1.64 ± 0.25 Å).

The value 0.20 *N* is a practical recommendation, not a property of the system, and we tested how much the SND3 alignment depends on it (SI Figure S2). Repeating the alignment at σ values interpolated to hit *n*_eff_/*N* between 0.05 and 0.90, and at uniform weights, shows that the absolute BRMSD tracks the choice directly, rising from 0.33 ± 0.06 Å at 0.05 *N* through 0.50 ± 0.08 Å at 0.20 *N* to 1.64 ± 0.25 Å at uniform weights, with no plateau. Across the σ values, the shape of the BRMSD time series remains unchanged. Correlating each series against the one at 0.20 *N* gives *r* = 0.94 at 0.10 *N* and *r* = 0.97 at 0.30 *N*; so conclusions about when the structure deviates from its average are insensitive to the target across that band, and only the scale of the metric shifts. The useful range is bounded on both sides. Above 0.5 *N*, the groove-to-rest weight ratio falls below two, reaching 0.98 at 0.90 *N*, and the metric no longer separates the rigid core from the mobile regions. Below 0.1 *N*, the alignment rests on a few hundred atoms, the time series decorrelates from the broader solutions (*r* = 0.65 at 0.05 *N*), and convergence to self-consistency slows markedly, from 31 iterations at 0.20 *N* to 115 and 152 at 0.05 *N* and 0.10 *N*. For other systems, we therefore suggest choosing the target so that *n*_eff_ comfortably exceeds the size of the substructure the alignment is meant to resolve, checking that the weight ratio still discriminates, and reporting the value used.

The weight optimization automatically concentrates weight on the TM groove without manual residue selection. The resulting BRMSD (0.50 ± 0.08 Å) at σ_op_ = 0.43 Å is ∼3 times smaller than the standard RMSD (1.64 ± 0.25 Å, uniform weights), corresponding to an approximately 10-fold reduction in variance. The domain-decomposed RMSD time series at σ_op_ (Figure 2B) shows that the TM-groove RMSD is substantially lower and less variable than both the non-groove domain (Rest) and the standard all-atom RMSD, and that the BRMSD itself lies below all three, confirming that weight concentration on rigid atoms suppresses the dominant source of conformational noise. Per-residue RMSF and atom weights at σ_op_, shown in SI Figure S3, demonstrate the exponential suppression of flexible domains. The BRMSD-aligned trajectory is shown in Movie S1 alongside the raw trajectory.

BRMSD uses no information about chain identity and thus applies to multi-subunit assemblies without modification. We tested this on the six-chain translocon complex from which SND3 was taken, aligning all 19,811 atoms with the same protocol (SI Figure S4). At σ_op_ = 0.77 Å, chosen by the same *n*_eff_ ≥ 0.20 *N* rule, the optimization places 99.7% of the weight on the SEC61 heterotrimer, with each of its three subunits enriched relative to their share of atoms (SEC61α 2.2×, SEC61γ 1.3×, SEC61β 1.2×), and essentially none on the accessory subunits CCDC47, SND3, and TRAPα. The rigid core of the assembly is therefore recovered across chain boundaries from the fluctuations alone. The BRMSD is 0.78 ± 0.08 Å against a standard RMSD of 2.93 ± 0.62 Å. Within SND3 the weight profile obtained in the complex differs strongly from the one obtained for the isolated chain (Jensen-Shannon distance *D*_JS_ = 0.90, defined in eq 26 below; SI Figure S4C). Analyzed alone, the optimization finds the internal TM groove of SND3; analyzed in the complex, SND3 atoms are instead weighted by their rigidity in the SEC61 frame, which is dominated by the rigid-body motion of SND3 relative to the channel. Which core is recovered is thus a property of the selection, consistent with the pool-invariance behavior examined below.

### 3.3 Focused Alignment

To demonstrate the focused alignment module, we selected TMD-1 in SND3. Although it is membrane-embedded and structurally ordered, it contributes marginally to the global alignment (*w*_TMD1_ = 0.002 at σ_op_) because its fluctuations are larger than those of TMD-2/3/4. Focused alignment recovers the internal dynamics of TMD-1 by explicitly biasing the weight optimization toward the TMD-1 atoms.

We scanned μ/θ ratio from 0 to 0.8 at σ = 0.43 Å (SI Figure S5). Figure 3A shows the per-residue normalized weight profiles for global alignment at σ_op_ (blue) and focused alignment at μ/θ = 0.53 (orange). The latter raises the per-residue weights sharply in TMD-1 while the TM-groove weight decreases and the non-groove rest remains suppressed. As μ/θ increases, *w*_TMD1_ fraction grows from 0.002 to 0.54 while the *w*_groove_ fraction decreases from 0.87 to 0.44 and *w*_rest_ fraction falls to 0.02 (Figure 3B). The TMD-1 mean RMSF decreases from 1.49 Å at global alignment (μ/θ = 0) to 1.14 Å with focused alignment at μ/θ = 0.53 (Figure 3C). This 24% reduction reflects the removal of rigid-body translations and rotations of TMD-1 relative to the rest of the protein. The per-residue weight and RMSF profiles for all μ/θ values are shown in SI Figure S5A.

**Figure 3:**
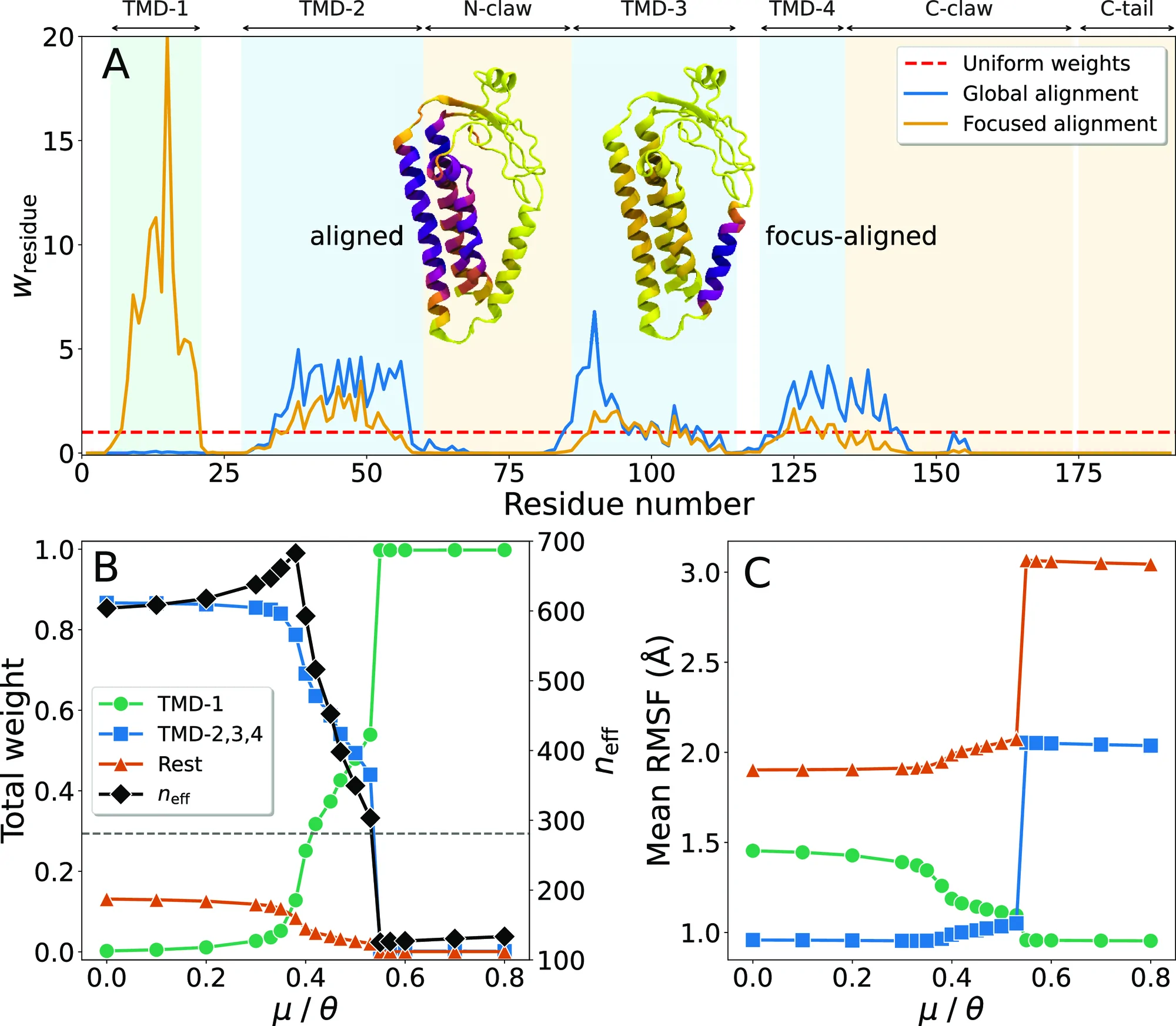
Results of focused alignment of SND3 onto TMD-1 are shown in three panels. (A) Per-residue weights are plotted against residue numbers. Global alignment at σ_op_ = 0.43 Å (blue) and focused alignment at (μ/θ)_op_ = 0.53 (orange) are shown simultaneously along with the uniform baseline (red dashed line). The average structures from each alignment are colored by the weights on each atom, with darker shades denoting higher weights. (B) Total weight fractions on TMD-1 (green), TM groove (blue), and the rest (orange) are plotted as functions of μ/θ. The effective atom count *n*_eff_ (black, right axis) and the TMD-1 size |*D*|= 281 (dashed) are overlaid. (C) Mean RMSF of each segment from the focus-aligned average structure is plotted against μ/θ.

The operating point (μ/θ)_op_ = 0.53 is identified from the *n*_eff_ criterion. At this value *n*_eff_ = 303, which just exceeds the TMD-1 atom count |*D*|= 281 (Figure 3B). At μ/θ = 0.55, *n*_eff_ drops below |*D*|(to 125), marking the onset of numerical over-concentration. Beyond the crossover at μ/θ ≈ 0.53, the focusing penalty overwhelms the entropy term and almost all weight collapses onto TMD-1 (*w*_TMD1_ → 1.0, *w*_groove_ → 0.002; Figure 3B). The alignment is then performed on TMD-1 alone. The now-unweighted TM groove and rest of the protein are no longer superimposed and their RMSF rises sharply (Figure 3C). This over-concentration, where *n*_eff_ < |*D*|, marks the upper usable limit of μ/θ. The per-μ/θ RMSF profiles in SI Figure S5B confirm that the groove and rest RMSF values are largely insensitive to the focusing parameter, isolating the TMD-1 RMSF reduction as a genuine alignment effect rather than an artifact.

### 3.4 Nonuniform Priors

We tested the two priors of Section 2.5 on the SND3 alignment at σ_op_ = 0.43 Å, using TMD-1 as the target domain so that the domain prior can be compared directly against the focusing penalty of the previous section.

The two routes behave differently, as the update rules predict. Sweeping μ/θ moves the weight on TMD-1 continuously, from *w*_TMD1_ = 0.002 at μ/θ = 0 to 0.539 at (μ/θ)_op_ = 0.53 (SI Figure S6B). Sweeping the prior mass *c* does not. Starting from *w* ^(1)^ = ***W***, the optimization stays on the groove solution up to *c* = 0.27, where *w*_TMD1_ = 0.012, and collapses onto TMD-1 at *c* ≥ 0.31, where *w*_TMD1_ = 0.999 (SI Figure S6C). No value of *c* reproduces the operating point of the focusing penalty, at which TMD-1 carries about half the weight while the rest of the protein is still superimposed.

The gap is not an artifact of the start point. Seeding the same fixed-point iteration from the converged groove solution and from the converged TMD-1 solution gives two branches that both remain stable across *c* = 0.21 to 0.35, with two co-existing solutions over that range. Their free energies cross at *c* = 0.254: below this value, the groove solution has the lower *G*, above it, the TMD-1 solution does. In the present case, the nonuniform prior thus produces two competing basins, resulting in hysteresis.

The algebra of Section 2.5 accounts for this. Evaluating the standard deviation of ln *w*_α_ + *d*_α_/*T* over α ∈ *D* identifies the effective concentration parameter that governs each update. For the prior route, this standard deviation is 9.4 × 10^–5^ at *T* = θ and 3.42 at *T* = θ + μ; for the focusing penalty it is 3.52 at *T* = θ and 8.1 × 10^–5^ at *T* = θ + μ. Each route is governed by its own effective parameter, which confirms that a prior cannot reproduce the focusing penalty. The consequence is visible within TMD-1 itself. Measuring the effective atom count (eq 12) of the weight distribution restricted to TMD-1 and renormalized, the focusing penalty gives 102 of |*D*|= 281 atoms against 95 for the collapsed prior and 75 for the uniform prior. The penalty thus spreads weight more evenly inside the domain. The prior states which atoms are expected to matter, while μ sets how uniformly they are weighted once selected.

The mass prior *W*_α_ ∝ *m*_α_ tests whether the optimal weights reduce to a conventional atom selection (SI Figure S7). They do not. Under the uniform prior, the 1548 hydrogen atoms make up 51.4% of the selection and carry 30.5% of the total weight, and the 389 backbone hydrogens alone carry 21.7%, more than the 191 C_α_ atoms at 12.9%. A C_α_ or heavy-atom selection would therefore discard atoms that the optimization weights heavily. Imposing the mass prior recovers the conventional picture, suppressing hydrogen to 3.3% and raising the heavy atoms from 69.5% to 96.7%, and it lowers the effective atom count of the solution. Mass weighting is thus available within the same framework as a prior, as a choice imposed on the analysis; however, the data here do not favor it.

### 3.5 Trajectory Smoothing

We smoothed the SND3 trajectory using a triangular window kernel, sweeping the half-width *W* over 5, 10, 20, 30, and 50 frames (full window 1 to 10.0 ns) at σ_op_ = 0.43 Å (SI Figure S8). We quantify the smoothing quality by the mean BRMSD of each raw frame from its locally windowed average. This quantity increases with window width because wider windows produce local averages further from any individual frame, ranging from 0.30 Å at *W* = 5 frames to 0.37 Å at *W* = 50 frames. We adopt *W*_op_ = 20 frames (4.0 ns full window). With the 4.0 ns full window below the slow TM-groove modulation time scale, sub-nanosecond fluctuations are smoothened without averaging out the slower collective motion. The mean BRMSD from the windowed average at *W*_op_ is 0.35 Å.

At *W*_op_, the mean BRMSD of the smoothed trajectory from the global average drops from 0.50 ± 0.08 Å (raw) to 0.35 ± 0.04 Å, a 30% reduction (SI Figure S9). The smoothed trajectory retains the slow TM groove modulations visible at time scales above 4 ns while suppressing sub-nanosecond fluctuations. Movie S2 shows the raw and smoothed trajectories side by side. Per-residue RMSF in SI Figure S10 shows that smoothing reduces fast fluctuations uniformly across the chain, while the slow, large-amplitude movements are retained.

### 3.6 Conformational Clustering

Unlike alignment and domain identification, conformational clustering of the open-to-closed transition requires a broad weight distribution rather than a concentrated one. The signal that separates the two states of ADK resides in the mobile NMPbind and LID domains. Concentrating weight on the rigid CORE (small σ) suppresses precisely these displacements and collapses the cluster centers onto a single point. A sweep of the alignment σ shows that the Calinski-Harabasz (CH) index (eq S1) is almost zero for σ ≲ 2 Å, where the cluster centers merge. The CH index rises sharply between σ = 3 and 4 Å and plateaus for larger σ. We adopt σ = 4.0 Å as the onset of this stable, high-CH regime (SI Figure S11).

To select τ, we swept over the values 1, 2, 5, 10, 20, and 50 Å^2^ at *K* = 2, evaluating the CH index (eq S1), minimum center separation (eq S2), and population balance (SI Figure S12). The CH index and center separation remain high for τ ≤ 5 Å^2^ and collapse to zero at τ ≥ 10 Å^2^, where the responsibilities become uniform and the two centers merge. We adopt the largest τ that preserves discrimination. At τ_op_ = 5.0 Å^2^, the two centers are well separated (center separation = 3.20 Å) and the partition is most balanced (|*q̅*_1_ – 0.5|= 0.02), whereas smaller τ over-sharpens toward hard *K*-means without improving the partition.

*K*-screening at τ_op_ identifies *K* = 2 as optimal. At *K* = 3, the minimum inter-center separation collapses to 0.0 Å across restarts, indicating fully degenerate centers. No third distinct conformational state was detectable by BRMSD clustering with these parameters.

The two clusters correspond to open-like (soft population 51.8%, hard count 10,610 frames) and closed-like (soft population 48.2%, hard count 9,081 frames) conformations (Figure 4). The corresponding cluster centers (responsibility-weighted average structures, eq 21) are located at *d*_NMPbind–LID_ = 26.2 Å, *d*_LID–CORE_ = 37.9 Å (open) and *d*_NMPbind–LID_ = 18.8 Å, *d*_LID–CORE_ = 33.7 Å (closed). They are denoted by the black circles in Figure 4A. The near equal populations reflect the pathway-sampling design of the DIMS ensemble and do not correspond to thermodynamic equilibrium free energies.^29^ DIMS trajectories sample structural coverage of the open-to-closed transition, not a Boltzmann distribution. Population fractions can thus not be interpreted as equilibrium state probabilities.

**Figure 4:**
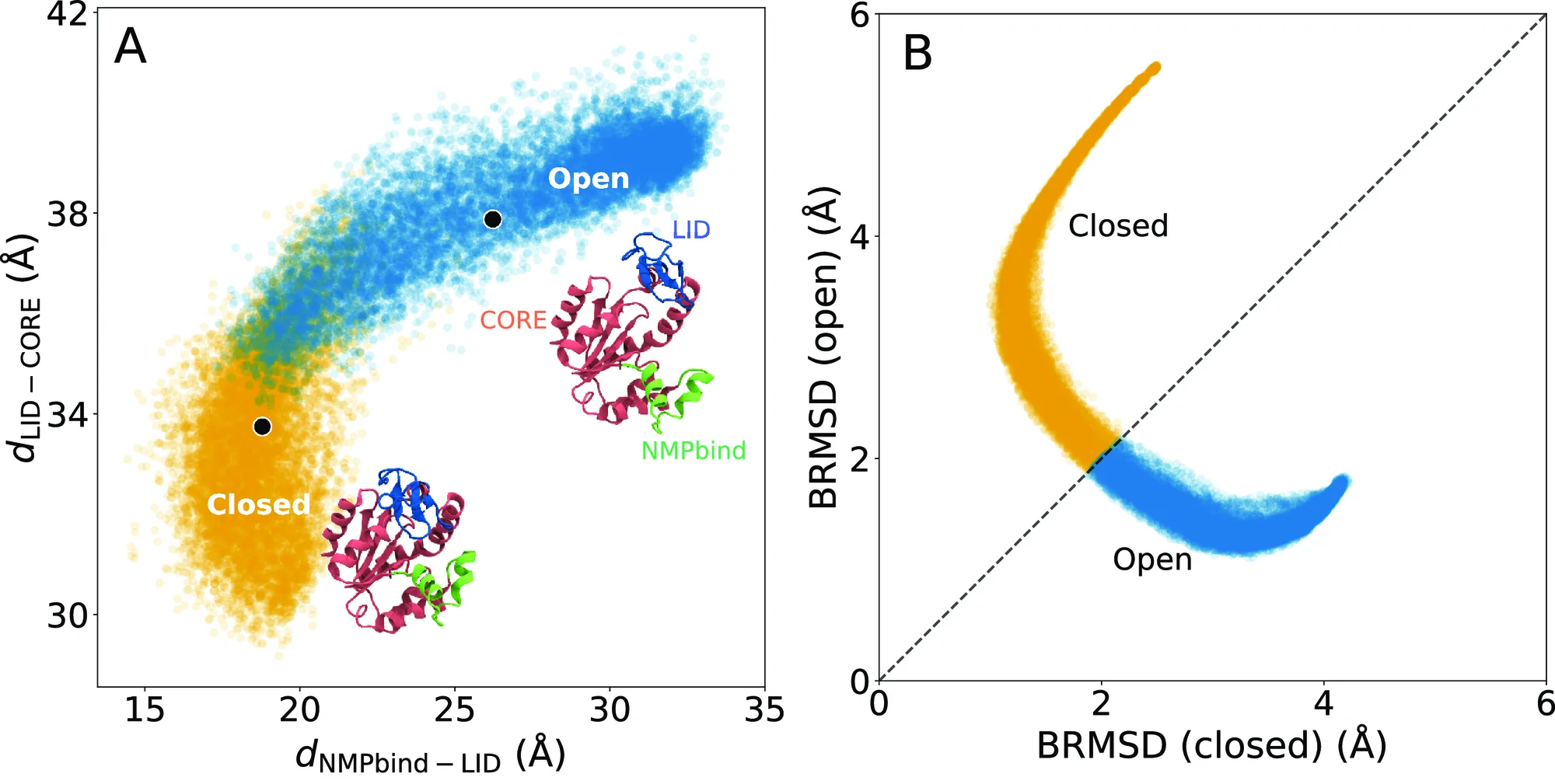
Conformational clustering of the 19,691-frame ADK DIMS ensemble (*K* = 2, τ_op_ = 5.0 Å^2^, σ = 4.0 Å). (A) Each frame placed by its order parameters *d*_NMPbind–LID_ and *d*_LID–CORE_, colored by hard cluster assignment: orange (closed) and blue (open). Black circles mark the two cluster centers, shown colored by domain (CORE red, LID blue, NMPbind green). (B) The same frames placed by their weighted BRMSD to the closed center (abscissa) and to the open center (ordinate), each computed with that cluster’s own weight vector. The dashed line marks equal BRMSD from both clusters.

Plotting each frame by its weighted BRMSD to the two cluster centers (Figure 4B), each distance evaluated in that cluster’s own weight vector, separates the ensemble into two off-diagonal arcs that meet only along the *d*_closed_ = *d*_open_ diagonal. Because every axis uses a cluster-adapted metric, a frame of one state measured against the other center is driven to large distance by precisely the mobile LID and NMPbind atoms that distinguish the two states, giving a cleaner separation than the geometric order parameters of Figure 4A. The diagonal crossing marks the transition dividing surface (*q* ≈ 0.5), and its low point density indicates rapid transit through the barrier region. The outward curvature of each arc reflects the arced, hinge-bending transition path between the open and closed end points rather than a straight interpolation between the two centers.

The responsibilities (*q*(*a*|*i*), where *a* is the cluster and *i* is the structure) correlate strongly with both order parameters. The Pearson correlation coefficients of *q*(cluster 1|*i*) with *d*_NMPbind–LID_ and *d*_LID–CORE_ are −0.88 and −0.94, respectively (SI Figure S13). The median of the ratio *d*_between_/*d*_within_ (between-cluster and within-cluster distances) is 2.48, and all frames have *d*_between_ > *d*_within_. This confirms that the two conformational states are genuinely distinct in BRMSD space (SI Figure S14). Per-residue backbone displacement between the two cluster centers (SI Figure S15) reveals the hierarchical displacement order, LID (mean 5.68 Å) > NMPbind (4.73 Å) ≫ CORE (1.11 Å), consistent with the ground-truth domain assignments.

### 3.7 Rigid Domain Identification

We first applied the domain identification module to SND3 to assess whether any sub-domain structure is resolvable. Sequential peeling recovers the domains when the alignment σ is set to the rigidity scale of TMD-1. A σ sweep locates the cleanest separation at σ = 1.0 Å (SI Figure S16), where round 1 recovers the TM groove and round 3 recovers TMD-1 as its own domain (SI Figure S17) with a Jaccard index of 0.44 to the TMD-1 reference (SI Figure S18). Round 2 falls on the cytosolic claws but does not form a clean domain (*J* = 0.28). The separation is sharp in σ. It peaks at 1.0 Å and collapses for σ ≥ 1.6 Å, where TMD-1 merges into the groove. This value matches the internal RMSF of TMD-1 measured by focused alignment (1.14 Å). Only the stiffest half of TMD-1 is recovered in round 3 because it is a partially rigid membrane-embedded helix rather than a fully independent domain.

For ADK, we applied sequential peeling to the 28,282-frame FRODA backbone ensemble. FRODA generates motions by propagating rigid-body constraints on covalent geometry, leaving its domain boundaries structurally unambiguous by construction. Hence, the domains are recovered more cleanly in ADK than SND3 (300 ns equilibrium run). However, simultaneous *K* = 3 optimization fails to detect the three different rigid domains in ADK. This happens because all three weight vectors collapse onto CORE regardless of the overlap penalty μ. Since the initial weights are uniform for every domain, the first iteration tends to concentrate all vectors onto the globally lowest-MSF region (CORE), from which the overlap-penalty gradient is too weak to escape. Sequential peeling resolves this by running three independent *K* = 1 optimizations on shrinking atom pools. The peeling order follows global rigidity. The most rigid domain is always found first. The order (CORE → LID → NMPbind) is thus data-determined rather than user-specified.

A σ sweep at *K* = 1 on the full 856-atom pool identifies σ_op_ = 3.0 Å as maximizing *J*_CORE_ (SI Figure S19). Here, *J*_*X*_ is the Jaccard score for domain *X*, which measures the overlap between the atoms recovered by the BRMSD module and the experimental ground truth. This criterion thus requires ground-truth domain labels. Figure 5 reports the recovered domains through the per-residue weight (the mean BRMSD weight of each residue’s backbone atoms). Each round is normalized by its own maximum, so that the most rigid residue in a round equals one. Round 1 (856 atoms, 5 restarts) recovers CORE with *J*_CORE_ = 0.94 and claims 616 atoms above the *t* = 0.50 threshold. Round 2 (240 non-CORE atoms, 5 restarts) identifies the LID domain (*J*_LID_ = 0.99), and Round 3 (96 remaining atoms, 5 restarts) recovers NMPbind with *J*_NMPbind_ = 1.00. The Jaccard scores for all three domains are given in SI Figure S20. At *t* = 0.50, the CORE mask absorbs 26 of the 120 NMPbind backbone atoms at the CORE-NMPbind boundary, but the remaining NMPbind and LID cores stay cleanly separated. The two flexible domains are thus recovered.

**Figure 5:**
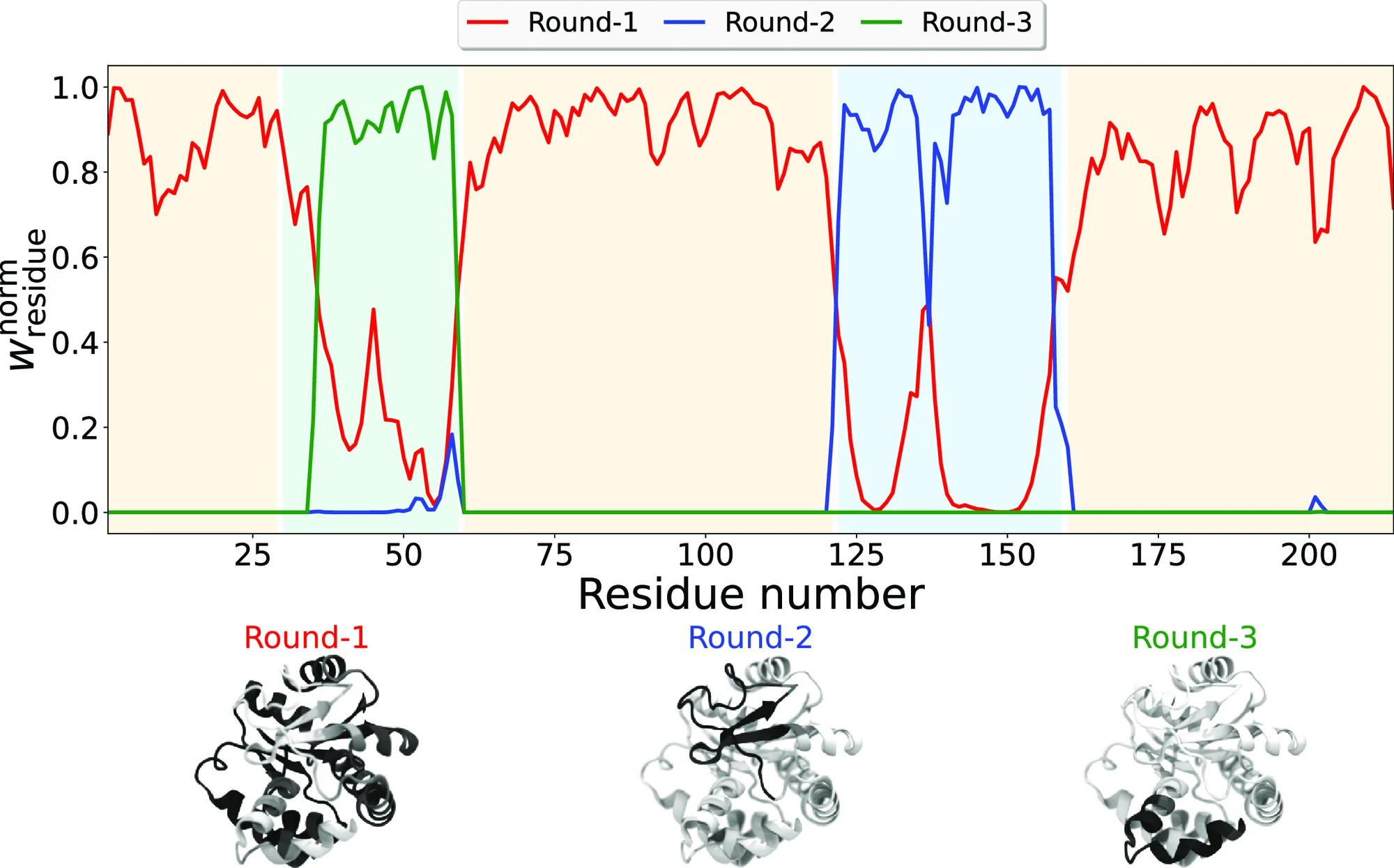
Rigid domain identification for ADK by sequential peeling on 28,282 FRODA backbone frames (856 atoms, σ_op_ = 3.0 Å). The per-residue weight profiles *w*_residue_^norm^ (mean atom weight of a residue, normalized within each round to its maximum) for the three peeling steps are overlaid: Round 1 (red, CORE), Round 2 (blue, LID), and Round 3 (green, NMPbind), with domain shading. The corresponding average structures, colored by weight, are shown below. Domains are detected in order of decreasing global rigidity.

A sensitivity sweep over *t* ∈ [0.40, 0.95] shows that *J*_CORE_ is flat (the step-1 optimization is independent of *t*), while *J*_LID_ and *J*_NMPbind_ degrade monotonically as *t* increases. For *t* ≤ 0.60, the CORE mask slightly overclaims (*N*_core_ = 580 to 636 vs. the ground-truth 584), but the non-CORE pool still separates cleanly into LID and NMPbind, giving *J*_LID_ ≥ 0.94 and *J*_NMPbind_ = 1.00. As *t* increases beyond 0.70, CORE is progressively underclaimed (*N*_core_ = 413 at *t* = 0.80, 201 at *t* = 0.90), leaving rigid CORE hinge residues in the pool that dominate the later steps and collapse both *J*_LID_ and *J*_NMPbind_ toward zero.

### 3.8 Consistency and Uniqueness of the Optimal Weights

Under the θ = *M*σ^2^ convention, each atom’s converged weight depends only on its own mean-square fluctuation relative to σ, independent of the number of frames *M* and of the number of atoms *N* in the selection. We verified both invariances on the SND3 alignment at σ_op_ = 0.43 Å (SI Figure S21). We quantify the difference between two normalized weight vectors ***w*** and ***w*** ′ by the Jensen-Shannon distance, the square root of the Jensen-Shannon divergence (a symmetrized form of the KL divergence).

26 $$D_{\mathrm{JS}}\left(w|w^{\prime }\right)={\left[\frac{1}{2\ln2}\sum_{\alpha }\left(w_{\alpha }\;\ln\frac{w_{\alpha }}{m_{\alpha }}+w\prime_{\alpha }\;\ln\frac{w\prime_{\alpha }}{m_{\alpha }}\right)\right]}^{1/2},,m_{\alpha }=\frac{1}{2}\left(w_{\alpha }+w\prime_{\alpha }\right)$$

*D*_JS_ is bounded in [0,1] and is well-defined even when some weights vanish. *D*_JS_ = 0 for identical weight vectors and *D*_JS_ = 1 for distributions with disjoint support.

Recomputing the weights from alternate frames (1501 of 3001 frames) leaves the per-residue weights unchanged (*D*_JS_ = 0.014), confirming *M* invariance (SI Figure S21A). Removing an entire mobile region from the selection and reoptimizing on the retained atoms similarly leaves their weights unchanged. Dropping TMD-1 (*D*_JS_ = 0.014) (SI Figure S21B) or the cytosolic claws (*D*_JS_ = 0.076) (SI Figure S21D) has almost no effect, whereas removing the rigid TM groove, which carries the dominant weight, forces a different solution, yielding a higher *D*_JS_ of 0.836 (SI Figure S21C). With the groove removed, the optimization does not spread weight evenly over the retained mobile atoms; it concentrates on the next most rigid substructure, and the effective atom count falls from 0.20 *N* to 0.11 *N*. The weights are thus invariant to random changes in the ensemble but are responsive to targeted changes.

Although *M*-invariance tests statistical thinning over a given time window, we assess convergence in time by optimizing the weights independently on the first and second halves of the trajectory (SI Figure S22A). These two solutions differ considerably, giving *D*_JS_ = 0.238 over all of the atoms. The disagreement sits almost entirely in the low-weight tail, where relative differences are large, but the contribution to the alignment is negligible. Restricted to the 700 atoms that carry 94% of the weight, the distance falls to 0.153, and for the 223 atoms carrying half the weight, it falls to 0.065. The atoms that determine the alignment are therefore reproduced far better than the full weight vector suggests, but the SND3 mean-square fluctuations are not converged at 150 ns.

The θ = *M*σ^2^ convention makes the sampling error of the weights directly accessible. Since the exponent of eq 9 reduces to *d*_α_/θ = MSF_α_/σ^2^, an error in the MSF propagates as

27 $$\delta \ln w_{\alpha }=-\frac{\delta {\mathrm{MSF}}_{\alpha }-\langle \delta \mathrm{MSF}\rangle_{w}}{\sigma^{2}},,{\left\langle \cdot \right\rangle }_{w}=\sum_{\beta }w_{\beta }{\left(\cdot \right)}_{\beta }$$

where the weighted mean enters through the normalization and removes the common mode. A block analysis then yields a per-atom error bar on the weights at negligible extra cost. Splitting the SND3 trajectory into ten blocks of 300 frames gives a median standard error of the MSF of 0.195 Å^2^, comparable to the value of σ^2^ = 0.183 Å^2^. On the atoms carrying above-average weight, the propagated δ ln *w*_α_ has median 0.12 against 0.21 for the spread observed when the weights are re-optimized independently on each block, with a correlation of 0.76 between the two (SI Figure S22B). The propagated value is the smaller of the two because it is a first-order estimate at a fixed reference structure, whereas re-optimization also lets the alignment respond. The uncertainty is lowest in the TM groove and one to two orders of magnitude larger in the mobile claws and termini (SI Figure S22C). As a result, the weights are best resolved where they are largest. Weights computed from the first fraction *f* of the trajectory approach the full-trajectory solution monotonically beyond *f* = 0.3, with *D*_JS_ falling from 0.255 at 30 ns to 0.020 at 270 ns and no clear plateau (SI Figure S22D). We therefore recommend reporting block error bars together with the weights and treating atoms whose δ ln *w*_α_ approaches unity as unresolved.

The weight update is a nonlinear fixed-point map, possibly with several competing self-consistent solutions corresponding to local minima of the free energy *G*. Seeding the SND3 alignment from 40 spatially localized weight vectors (Gaussian bumps of half-width 10 Å centered on random atoms) and clustering the converged solutions by *D*_JS_ shows that, at the operating point σ_op_, all initializations converge to a single solution (SI Figure S23). As σ decreases below σ_op_, the landscape fragments into multiple competing minima (SI Figure S23A), each concentrating weight on a different local rigid substructure (SI Figure S23C), and all with higher *G* than the global minimum (SI Figure S23B). This justifies the single initialization used for the alignment and smoothing modules at σ_op_. It also explains why clustering and domain identification use random restarts.

## 4 Conclusions

BRMSD unifies multiple structural analysis tasks within a single Bayesian framework. This includes global alignment, focused alignment, trajectory smoothing, conformational clustering, and rigid domain identification. All five use a free-energy functional with a concentration parameter σ (via θ = *M*σ^2^), which establishes the MSF threshold such that atoms with fluctuations well below the σ scale are assigned near-uniform weights, effectively preventing the metric from focusing on local noise. The optimal σ is module- and system-specific. Parameter selection follows the same diagnostic in each module, monitoring *n*_eff_ or a module-specific quality index as a function of σ. Because the weights are set by the per-atom MSF, they are insensitive to trajectory length once the MSF estimates have converged. For short trajectories, where the per-atom MSF has not yet converged, σ_op_ and the recovered weight profiles should be treated as provisional.

BRMSD was benchmarked against two well-resolved systems. In SND3, alignment at σ_op_ = 0.43 Å reduces the apparent RMSD threefold relative to standard backbone superposition, automatically suppressing mobile cytosolic claws without manual residue selection. Focused alignment onto TMD-1 at (μ/θ)_op_ = 0.53 reduces the TMD-1 RMSF by a further 24%, revealing its internal dynamics against the background of TM groove motion. Smoothing with a 4 ns triangular window reduces BRMSD variance by ∼73% while preserving slow TM groove modulations.

In ADK, soft *K*-means clustering with cluster-specific weight vectors recovers the known open/closed bimodality. The cluster centers (responsibility-weighted average structures) align with the expected order-parameter regions of the conformational landscape. Sequential domain detection recovers all three known ADK domains in order of decreasing global rigidity (CORE, LID, and NMPbind). For SND3, simultaneous optimization finds no sub-domain partition, and sequential peeling at σ = 1.0 Å partially recovers TMD-1 as a separate rigid unit, at the same fluctuation scale identified by focused alignment.

The contribution of BRMSD is to automate, reproducibly and within a single framework, the atom weighting that one would otherwise assign manually. A small σ recovers a rigid core without manual selection, whereas a large σ recovers the standard RMSD metric where the discriminating signal is distributed. However, in its current form BRMSD has certain limitations, as listed below.

1. The operating point σ is task-dependent and is not governed by a single universal rule. The converged weights depend on per-atom MSF convergence, and this dependence is not weak: for SND3 the weights from the two halves of a 300 ns trajectory still differ by *D*_JS_ = 0.238 over all atoms, although the atoms carrying most of the weight agree considerably better (SI Figure S22). Weight profiles from short trajectories should be treated as provisional and reported with the block error bars of eq 27.
2. For conformational clustering and rigid domain identification, the operating parameters σ_op_, τ_op_, and the peeling threshold *t* were selected using quality indices (CH and Jaccard) that reference the known cluster or domain assignments. A fully label-free selector for these parameters remains an open problem.
3. The current implementation of BRMSD is CPU only.
4. The Mahalanobis-like extension for anisotropic fluctuations (eq 24) is derived but not yet implemented.

Beyond the soft *K*-means module implemented here, the BRMSD metric is a drop-in replacement for the RMSD in any distance-based clustering algorithm. Once the weights ***w*** have been determined by a single alignment pass, eq 1 defines a pairwise distance MSD­(x_*i*_,*x*_*j*_; ***w***)^1/2^ between any two frames that can be substituted for the unweighted RMSD in, for example, GROMOS-style neighbor-count clustering,^30^ hierarchical linkage, or *K*-medoids. The only additional cost is the one-time weight optimization; the pairwise distance evaluation itself is no more expensive than the standard RMSD. Most traditional clustering protocols rely on a numerically convenient metric chosen a priori. In the BRMSD analysis, one instead learns an optimal metric from the data, given a model of the fluctuations, which should prove to be useful in the clustering of conformations.

The current implementation uses a self-consistent fixed-point iteration to find the entropy-optimal weights. The converged BRMSD weights could serve as an atom-selection mask for time-lagged independent component analysis (TICA).^31,32^ Restricting the coordinate input to atoms with *w*_α_ above a threshold removes noise-dominated degrees of freedom before slow-mode identification, which should improve MSM-based kinetic models.^33^

Training a neural network to predict per-atom weights from local structural features, using the maximum-entropy loss *G* as the training objective, would bypass the fixed-point iteration and make the method applicable to large ensembles at low marginal cost. Related work has combined neural networks with maximum-entropy principles for active learning of conformational ensembles,^34^ where maximum entropy guides the choice of which conformations to simulate next. Here, we instead propose using *G* as a training loss for predicting what weights to assign, a complementary application of the same principle. Further directions include GPU-accelerated kernels for large ensembles (*M* ≳ 10^5^ or *N* ≳ 10^4^), implementing the Mahalanobis-like PCA-mode weighting of eq 24, and a quantitative, label-free σ selector for clustering and domain detection.

## Supplementary Material







## Data Availability

The BRMSD package is openly available at https://github.com/bio-phys/BRMSD under the MIT license. The MD simulation files of SND3 are available on Zenodo (16745188).^20^ The ADK trajectories (DIMS and FRODA) are distributed through the MDAnalysisData repository.^24^

## Notes

Raw and BRMSD-aligned SND3 trajectories (MP4)
Raw and smoothed SND3 trajectories (MP4)
Clustering and domain detection quality metrics; implementation details; computational timing of all benchmark runs (Table S1); Lorenz curves of the SND3 weights (Figure S1); sensitivity of the SND3 alignment to the *n*_eff_ target (Figure S2); per-residue RMSF and weights of SND3 (Figure S3); BRMSD alignment of the six-chain translocon complex (Figure S4); weight and RMSF profiles of SND3 focused alignment (Figure S5); nonuniform domain prior versus focusing penalty (Figure S6); mass prior versus uniform prior (Figure S7); SND3 smoothing window sweep, time series, and per-residue RMSF (Figures S8–S10); ADK clustering quality versus σ and τ (Figures S11 and S12); ADK cluster responsibilities, within- and between-cluster distances, and per-residue cluster-center displacements (Figures S13–S15); SND3 sequential peeling σ selection, per-residue weights, and Jaccard indices (Figures S16–S18); ADK rigid-domain detection σ sweep and Jaccard indices (Figures S19 and S20); consistency of the SND3 weights (Figure S21); finite-sampling uncertainty of the SND3 weights (Figure S22); and fixed-point weight solutions of the SND3 alignment (Figure S23) (PDF)
