# Supplementary material for: Bayesian Learning of Distance Metrics Beyond RMSD for Biomolecule Alignment, Clustering, and Domain Identification
Source: J Chem Theory Comput. 2026 Oct 1;22(19):10475–87. doi: 10.1021/acs.jctc.6c01395 (PMC13671183; doi:10.1021/acs.jctc.6c01395)
Supplement: Supplementary file 1 [file ct6c01395_si_003.pdf]

Supporting Information: Bayesian Learning of Distance Metrics Beyond RMSD for Biomolecule Alignment, Clustering, and Domain Identification

Saumyak Mukherjee[†,‡] and Gerhard Hummer[*,†,¶]

†*Theoretical Biophysics, Max Planck Institute of Biophysics, 60438 Frankfurt am Main, Germany*

‡*Department of Chemical Sciences, Indian Institute of Science Education and Research, Berhampur, 760003 Odisha, India*

¶*Institute of Biophysics, Goethe University Frankfurt, 60438 Frankfurt am Main, Germany*

E-mail: gerhard.hummer@biophys.mpg.de

Supporting Information Text

Clustering and Domain Detection Quality Metrics

Calinski-Harabasz (CH) Index

The CH index[S1] measures the ratio of between-cluster to within-cluster scatter.

$$\mathrm{CH}(K) = \frac{B(K)/(K-1)}{W(K)/(M-K)}, \tag{S1}$$

where M is the number of frames, $B(K)$ is the total between-cluster scatter (sum of cluster-size-weighted squared distances from K^{th} cluster centroid to the global centroid), and $W(K)$ is the total within-cluster scatter (sum of squared distances from each frame to its assigned cluster centroid), both computed in BRMSD space. A higher CH indicates clusters that are simultaneously more compact and better separated.

Minimum Inter-centre Separation

The minimum squared Euclidean distance between any two cluster centres in BRMSD space,

$$d^2_{\min}(K) = \min_{i \neq j} \mathrm{MSD}(s_i, s_j; \boldsymbol{w}), \tag{S2}$$

detects degenerate solutions in which two centres collapse onto the same point. At $K = 3$ for the ADK ensemble, $d_{\min} = 0.0$ Å, confirming that the third centre has no distinct structural identity. This check is necessary because CH can be undefined or misleading when $W(K) \approx 0$ for a degenerate cluster.

Population Balance

For $K = 2$, the scalar $|\bar{q}_1 - 0.5|$ measures the deviation of the cluster-1 population fraction $\bar{q}_1 = M^{-1} \sum_i q(1|i)$ from an equal split, where $q(1|i) \in [0, 1]$ is the soft responsibility of frame i for cluster 1. A value near zero indicates a balanced partition, and a value near 0.5 indicates that one cluster absorbs nearly all frames. We report this metric alongside CH because a high-CH solution that is also severely imbalanced ($|\bar{q}_1 - 0.5| \gtrsim 0.3$) may reflect a single dominant state with outliers rather than two physically distinct conformations. At $\tau_{\mathrm{op}} = 5.0$ Å^2, $|\bar{q}_1 - 0.5| = 0.02$, confirming a near-equal open/closed partition.

Jaccard Index

$$J(A, B) = \frac{|A \cap B|}{|A \cup B|}, \tag{S3}$$

where A is the set of atoms in the recovered domain (binarized at $w_{k\alpha} > t \cdot \max_\alpha w_{k\alpha}$) and B is the ground-truth domain atom set. $J = 1$ indicates perfect recovery, and $J = 0$ indicates no overlap. We use $t = 0.50$ and report domains with $J > 0.5$ as successfully recovered.

Implementation

All five modules are implemented in the open-source Python package `BRMSD`. The package uses NumPy for numerical kernels, MDAnalysis[S2,S3] for trajectory I/O and atom selection, and joblib for optional multi-threaded parallelism over restarts. Each module exposes a self-contained function (`BRMSD_iterate`, `BRMSD_focus_iterate`, `BRMSD_smooth`, `soft_kmeans`, `identify_domains_sequential`) with a common interface: coordinate array, σ value, and module-specific hyperparameters (W, μ/θ, τ, K, μ, n_r). Python 3.10+ and MDAnalysis 2.x are required.

For the alignment and smoothing modules, a single initialization from the first frame ($s^{(1)} = x_1$, $w_\alpha^{(1)} = W_\alpha$) is used. Convergence is declared when the per-iteration changes in both the average structure and the atom weights fall below the tolerance `tol` (default 10^{-3}), that is $\Delta_{\text{struct}} <$ `tol` and $\Delta_{\text{weights}} <$ `tol`. For clustering and domain identification, n_r restarts are run in parallel using joblib, each initialized with random center selection. The restart with the lowest converged G is retained. For the benchmarks reported here, $n_r = 3$ restarts are used for clustering and $n_r = 5$ for domain identification. In both cases, the selected solution was reproduced in at least two of the n_r runs, confirming reproducibility of the reported optima.

The per-iteration computational cost scales as $\mathcal{O}(MN)$ for the weight update and $\mathcal{O}(N^3)$ for the Kabsch rotation, where M is the number of frames and N the number of atoms in the selection. Practical runtimes are summarized in Table S1. Alignment of the SND3 trajectory ($M = 3001$, $N = 3013$ all-atom) converges in 31 iterations. Smoothing adds a multiplicative factor of M over alignment but benefits from multi-process parallelism over frames via joblib. Focused alignment converges similarly to standard alignment at each μ/θ value. Clustering

and domain detection use joblib parallelism over restarts. Systems with substantially larger frame counts ($M \gtrsim 10^5$) or atom selections ($N \gtrsim 10^4$) would benefit from GPU-accelerated matrix operations, which the current implementation does not support.

Supplementary Tables

Table S1: Computational timing for all benchmark runs on a single CPU core unless otherwise noted (Python 3.12, AMD EPYC server node).

Module	System	M	N	Restarts	Wall time
Alignment	SND3	3001	3013	1	52 s
Focused align.	SND3	3001	3013	1 per μ/θ	50 s each
Smoothing	SND3	3001	3013	1	349 s (parallel)
Clustering	ADK DIMS	19691	3341	3 (×3 threads)	1307 s
Domain (step 1)	ADK FRODA	28282	856	5 (×3 threads)	316 s
Domain (step 2)	ADK FRODA	28282	240	5 (×3 threads)	165 s
Domain (step 3)	ADK FRODA	28282	96	5 (×3 threads)	73 s

Supplementary Figures

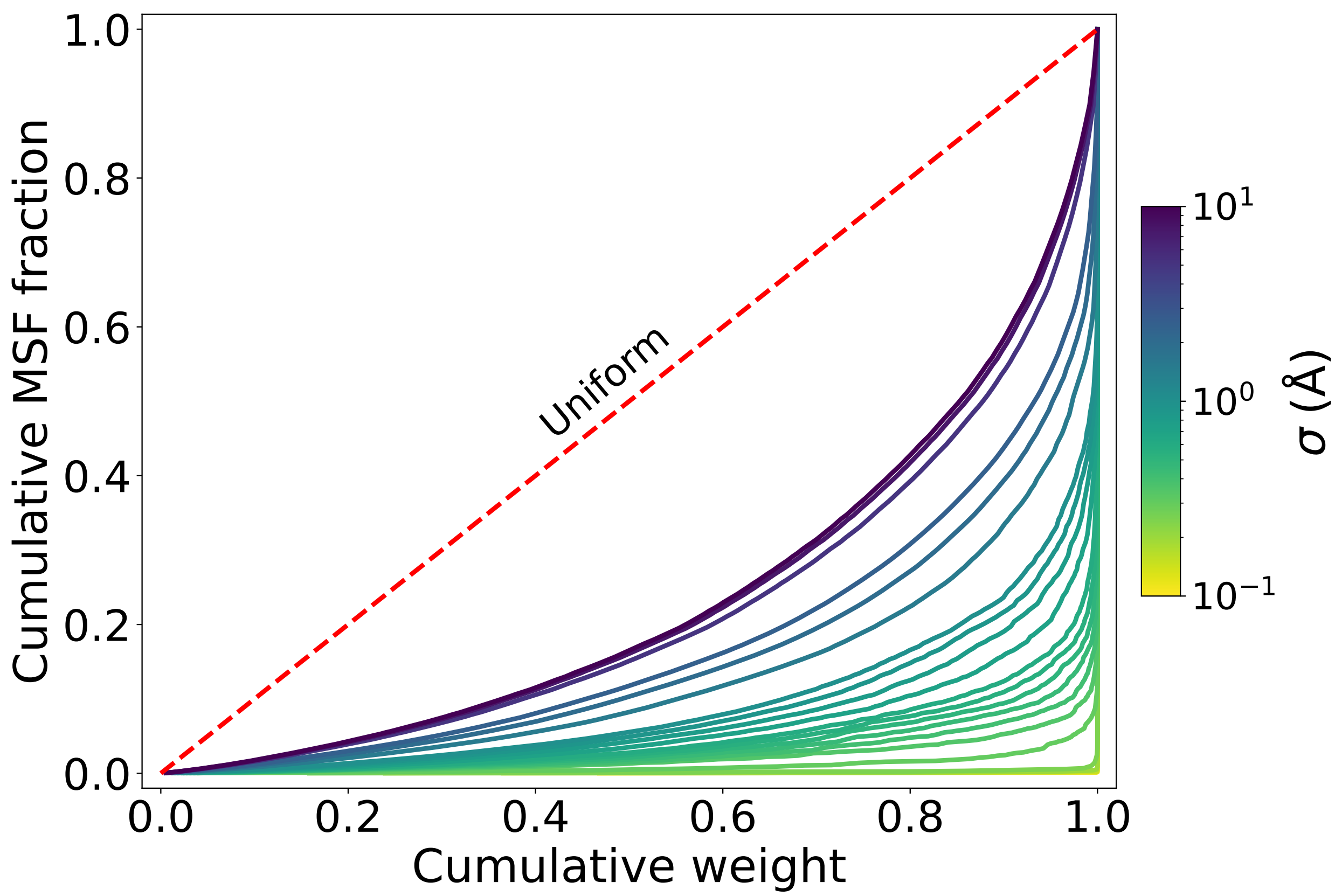

Figure S1: The Lorenz curve (cumulative MSF fraction versus cumulative atom weight) for SND3 is plotted at $\sigma = 0.1$ to 10.0 Å. As σ decreases, the curves depart progressively from the diagonal (equal weights on all atoms), quantifying how weight becomes concentrated on low-MSF (rigid) atoms. At the lowest σ values, the most-rigid atoms carry most of the total weight.

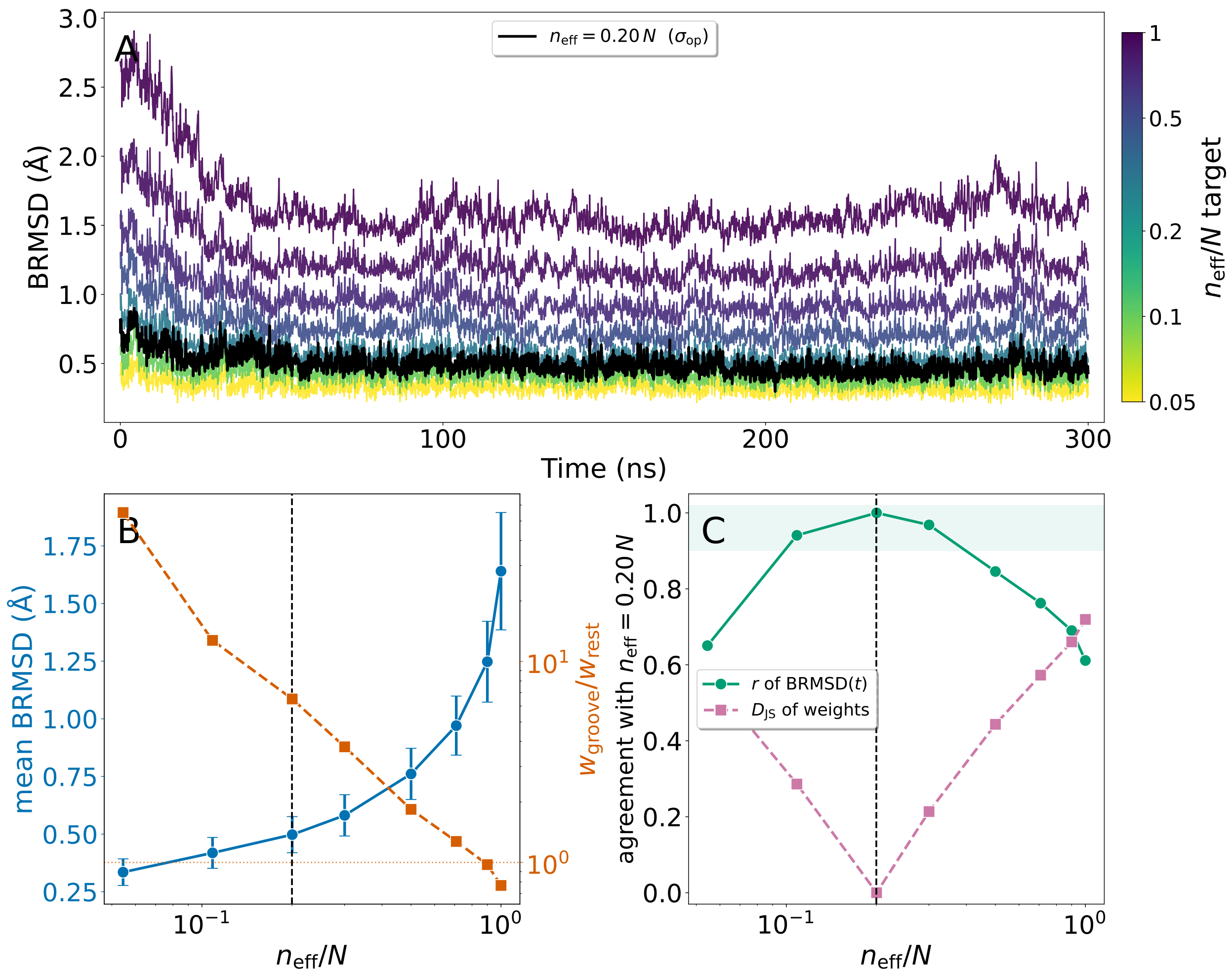

Figure S2: Sensitivity of the SND3 alignment to the n_{eff} target that fixes σ_{op}. (A) BRMSD time series obtained at σ values interpolated to reach n_{eff}/N between 0.05 and 1.00, colored by target (colorbar). The solution at the adopted target $n_{\mathrm{eff}} = 0.20\,N$ is drawn in black. Except for a change in magnitude, the curves keep a similar temporal structure. (B) Mean BRMSD with standard deviation (blue, left axis) and the weight ratio $w_{\mathrm{groove}}/w_{\mathrm{rest}}$ (orange, right axis, logarithmic) against the achieved n_{eff}/N. The dotted line marks equal weighting of groove and rest, and the dashed vertical line the adopted target. (C) Agreement with the $0.20\,N$ solution, measured by the Pearson correlation of the BRMSD time series (green) and the Jensen-Shannon distance between weight vectors (purple). The shaded band marks $r \geq 0.9$, which spans roughly $0.1\,N$ to $0.3\,N$.

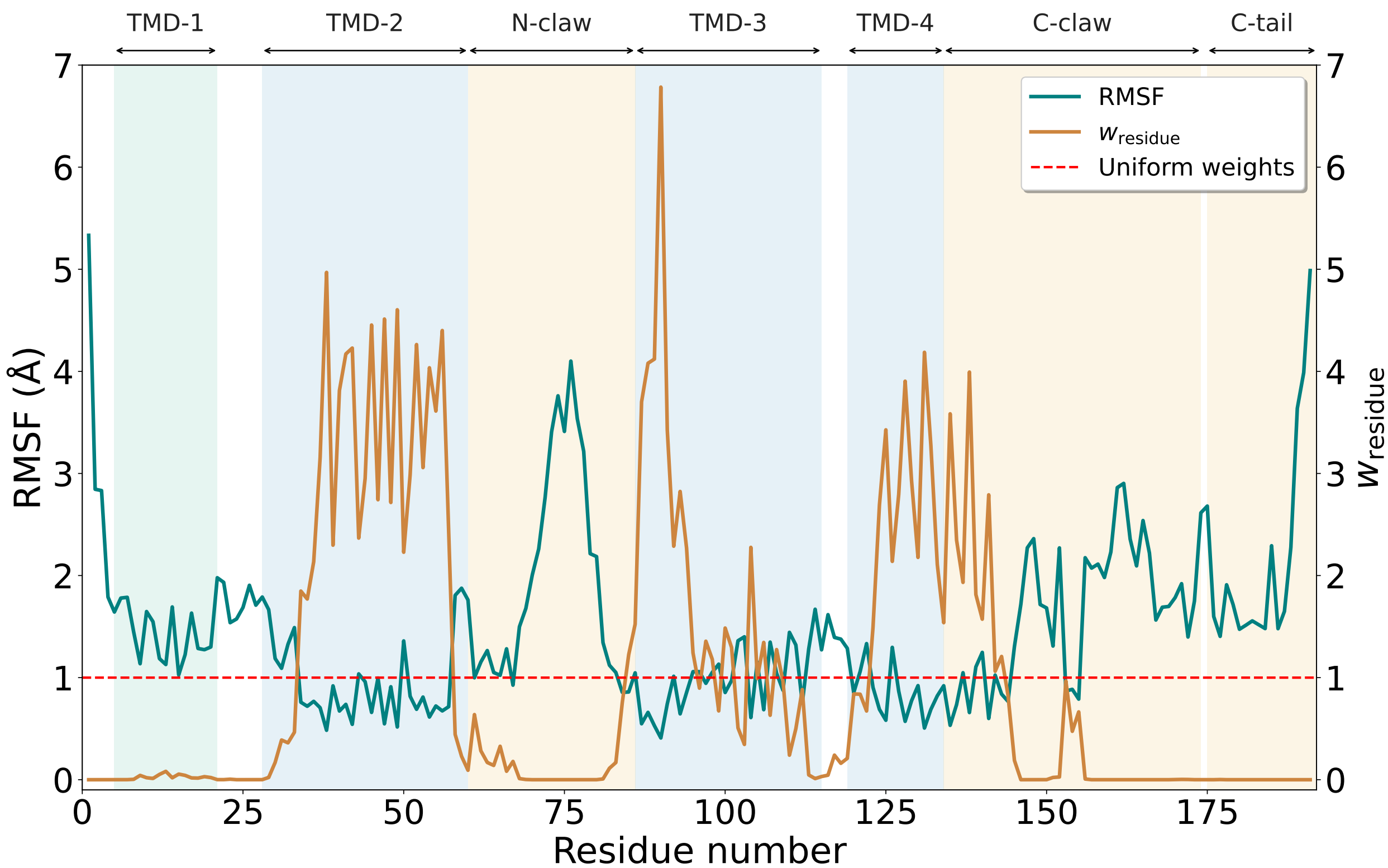

Figure S3: Per-residue RMSF (teal, left axis) and per-residue weight w_{residue} (brown, right axis) at $\sigma_{\mathrm{op}} = 0.43$ Å for SND3 are plotted as functions of residue number. Domain shading indicates TMD-1 (green), the TM groove TMD-2/TMD-3/TMD-4 (blue), and the mobile N-claw/C-claw/C-tail (orange). Weights and RMSF are anticorrelated, resulting in exponentially suppressed weights of high-RMSF residues.

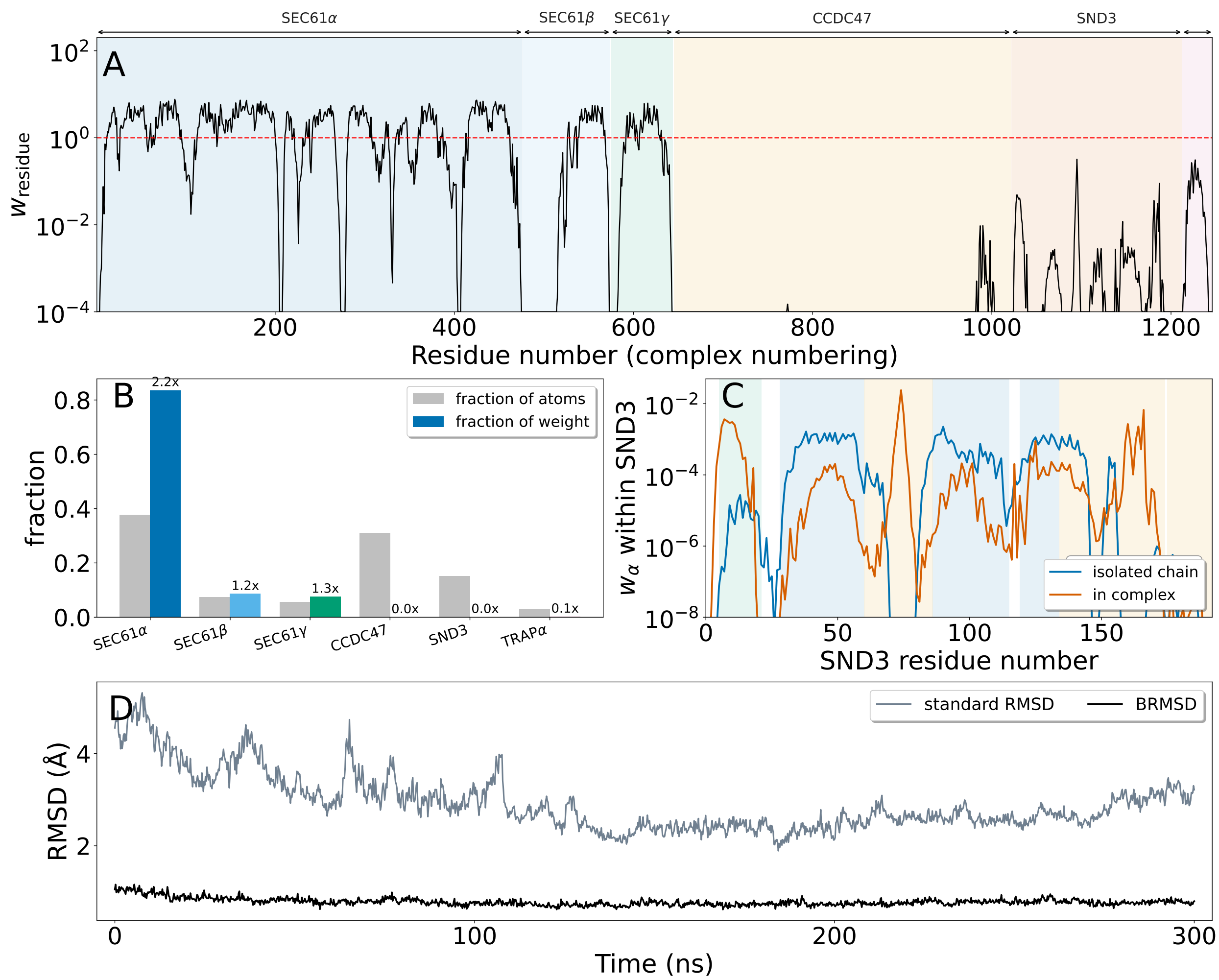

Figure S4: BRMSD applied to the six-chain translocon complex (19811 atoms, 1501 frames at 200 ps spacing, $\sigma_{\text{op}} = 0.77$ Å). (A) Per-residue weight w_{residue} along the complex on a logarithmic scale, with the chains shaded and labelled and the red dashed line marking uniform weighting. (B) Fraction of atoms (gray) against fraction of the optimized weight (colored) for each chain; the number above each bar is the enrichment, the ratio of the two. (C) Weights within SND3, renormalized over its atoms, obtained from the complex (orange) and from the isolated chain (blue); domain shading follows Fig. S3. (D) BRMSD and standard RMSD time series for the complex. In seven frames of the deposited trajectory the SEC61β chain is wrapped one periodic image away from the rest of the assembly. Each chain is individually whole, and these frames were repaired before analysis by shifting every chain to its minimum image about the SEC61α centroid.

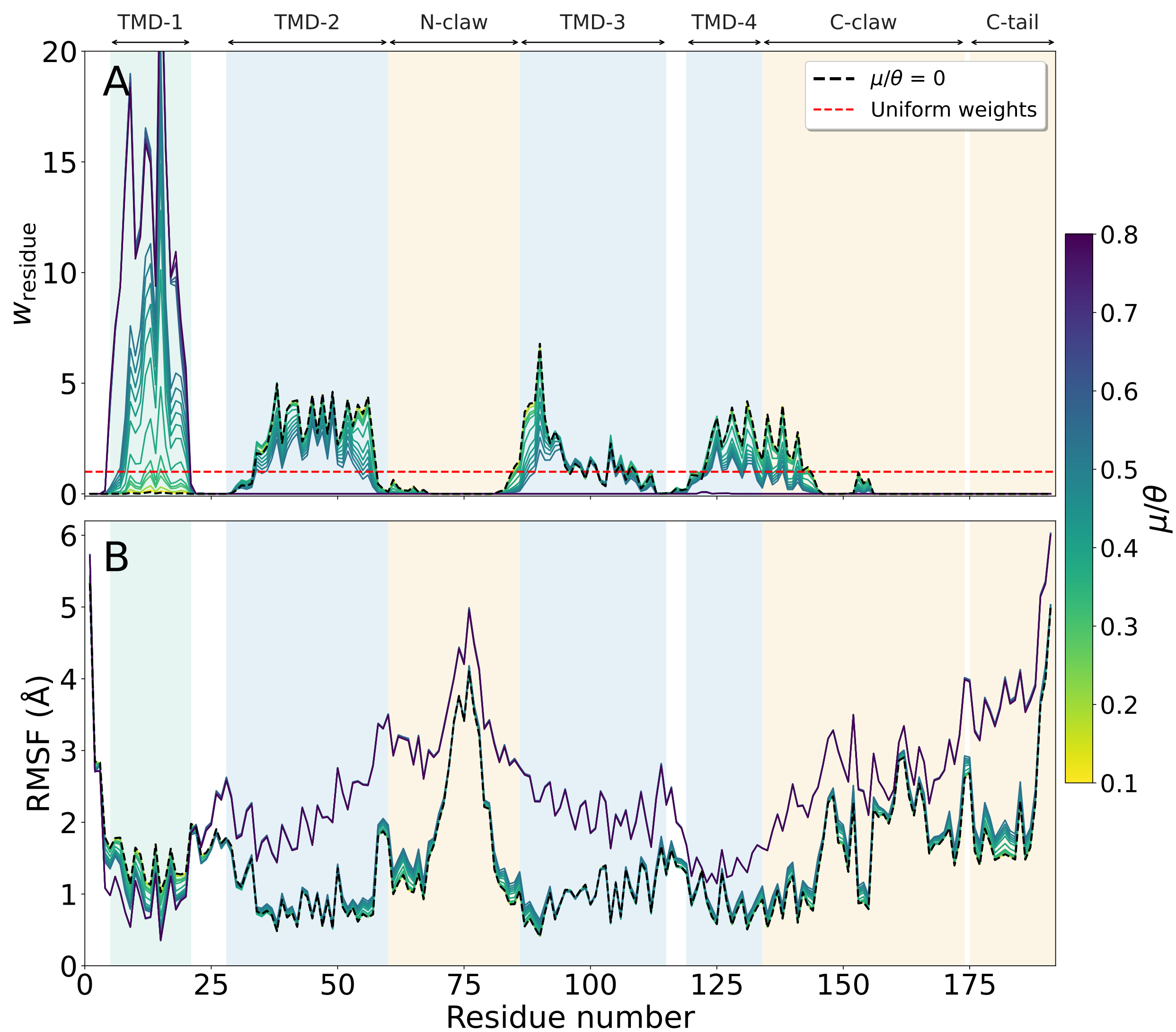

Figure S5: Weight and RMSF profiles for SND3 focused alignment onto TMD-1 are shown for $\mu/\theta = 0$ to 0.8. (A) Per-residue weights w_{residue} as a function of residue number. The TMD-1 (green shading) weights grow progressively as μ/θ increases from the global-alignment baseline (blue) to the operating point (orange, $(\mu/\theta)_{\mathrm{op}} = 0.53$). (B) Per-residue RMSF as a function of residue number for all μ/θ values. The RMSF is insensitive to μ/θ, confirming that the weight redistribution reflects the focusing constraint rather than changes in atomic fluctuations.

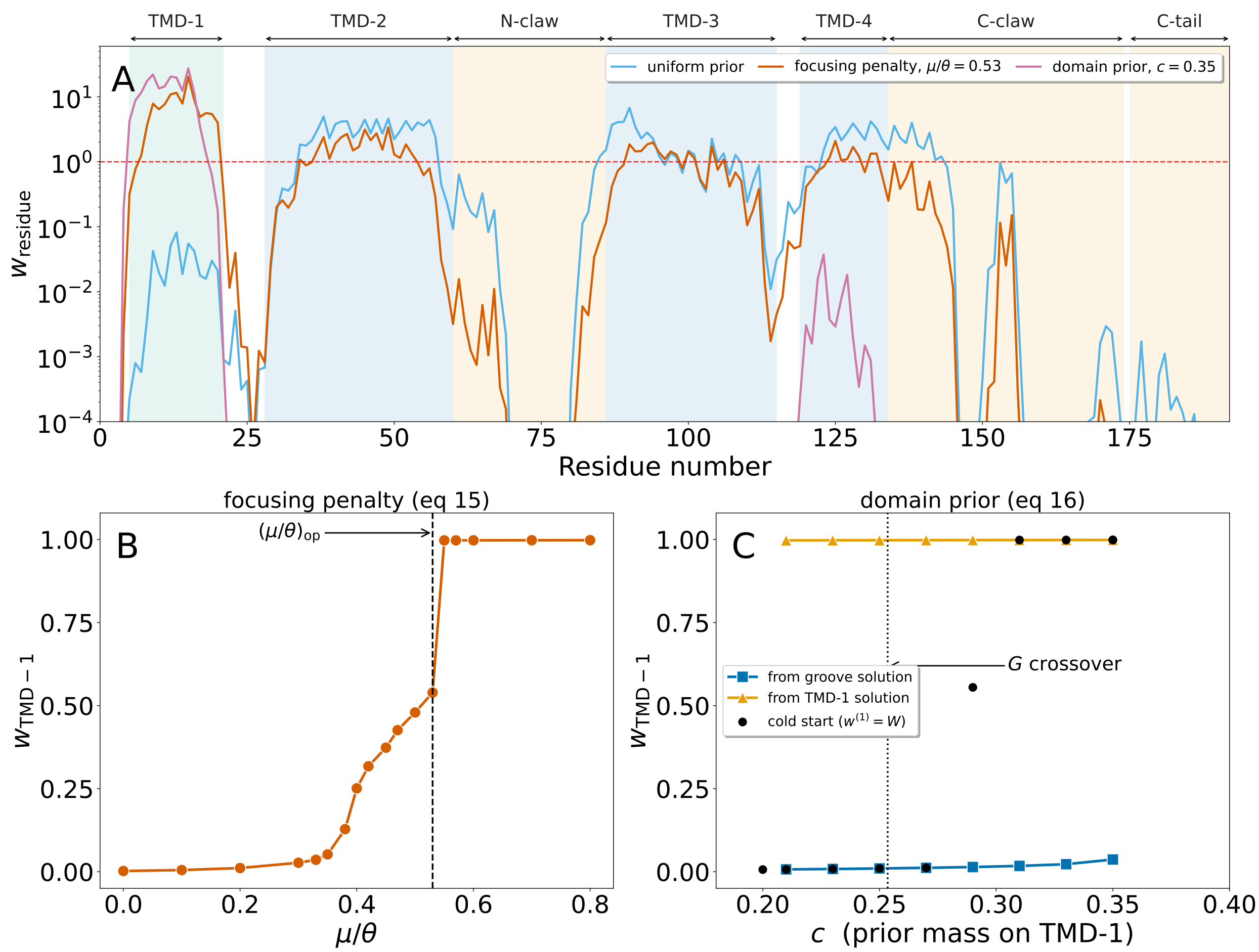

Figure S6: Nonuniform domain prior compared with the focusing penalty, for SND3 alignment onto TMD-1 at $\sigma_{\mathrm{op}} = 0.43$ Å. (A) Per-residue weights w_{residue} on a logarithmic scale for the uniform prior (blue), the focusing penalty at $(\mu/\theta)_{\mathrm{op}} = 0.53$ (orange), and the domain prior at $c = 0.35$ (purple). The red dashed line marks uniform weighting and the domain shading follows Fig. S3. (B) Total weight on TMD-1 as a function of μ/θ. The penalty moves the weight continuously up to the operating point (dashed line) before the solution collapses onto TMD-1. (C) Total weight on TMD-1 as a function of the prior mass c, for iterations started from the prior itself (black), from the converged groove solution (blue squares), and from the converged TMD-1 solution (orange triangles). The two branches coexist over the whole range, and their free energies cross at $c = 0.254$ (dotted line). The prior selects a basin, whereas the penalty tunes the weights.

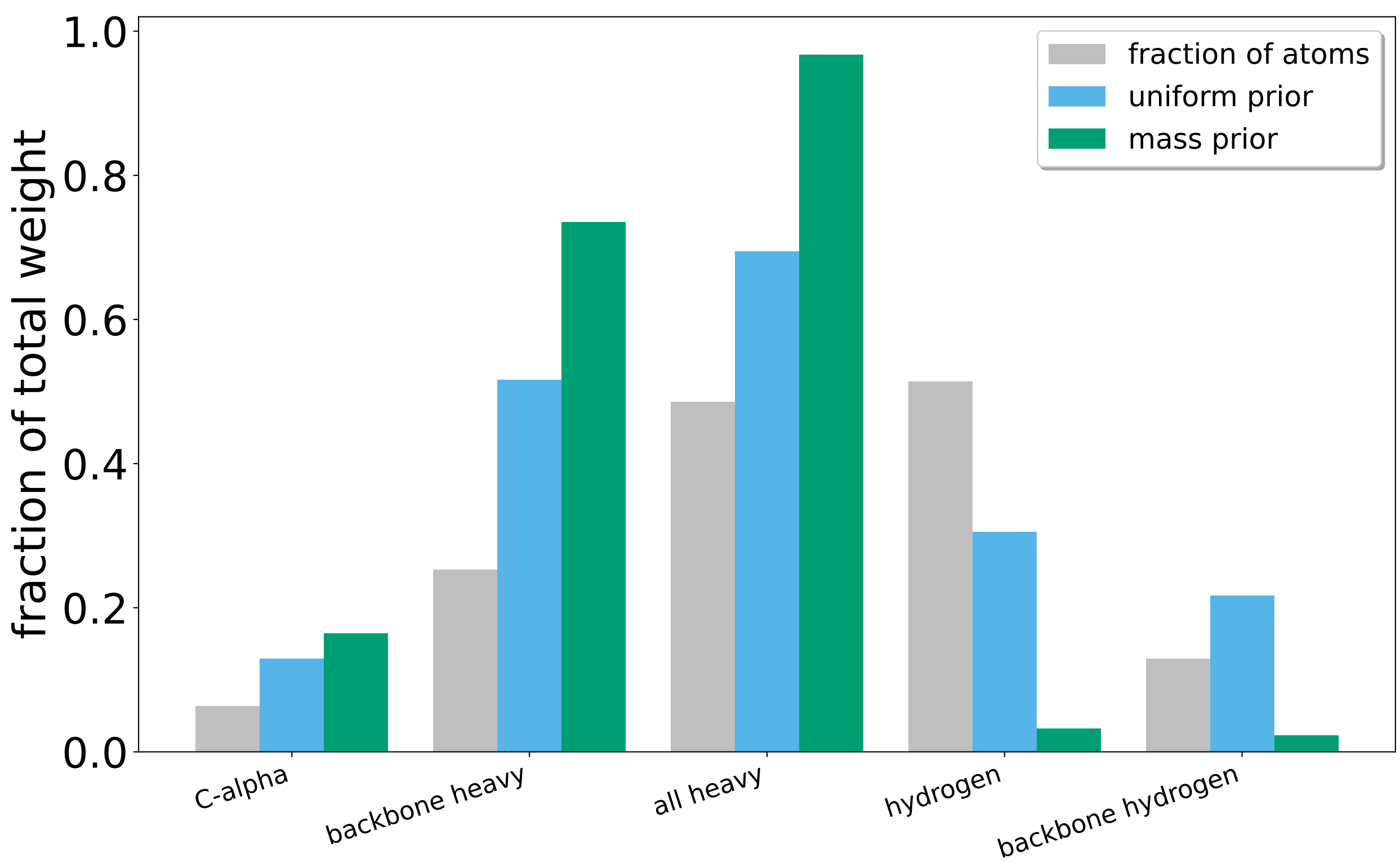

Figure S7: Mass prior $W_\alpha \propto m_\alpha$ compared with the uniform prior for SND3 at $\sigma_{\text{op}} = 0.43$ Å. Bars give the fraction of atoms in each class (gray) and the fraction of the total optimized weight under the uniform prior (blue) and under the mass prior (green). Under the uniform prior, the backbone hydrogen atoms carry more weight than the C_α atoms. The Bayes-optimal weights thus do not normally reduce to a conventional C_α or heavy-atom selection. The mass prior suppresses favors heavy-atom weighting.

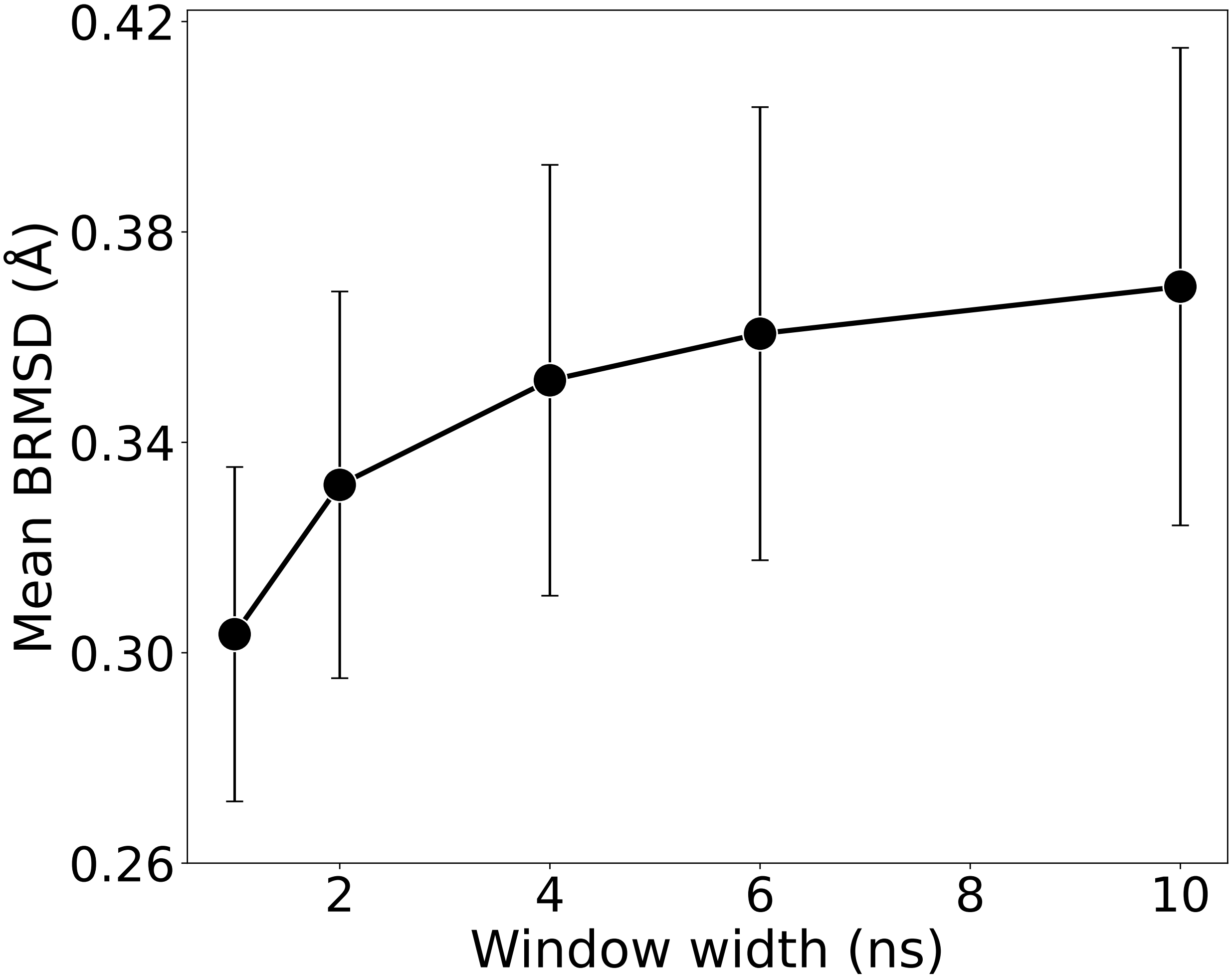

Figure S8: For SND3 smoothing, the mean BRMSD deviation of each raw frame from its locally windowed average is plotted versus total window width (2× half-width, ns). Points at full window widths 1, 2, 4, 6, and 10 ns correspond to half-widths $W = 5, 10, 20, 30$, and 50 frames. The mean deviation increases monotonically from 0.30 Å to 0.37 Å as wider windows produce local averages further from any individual raw frame. The operating point $W_{\mathrm{op}} = 20$ (4 ns) is marked by a dashed line. Error bars represent standard deviation.

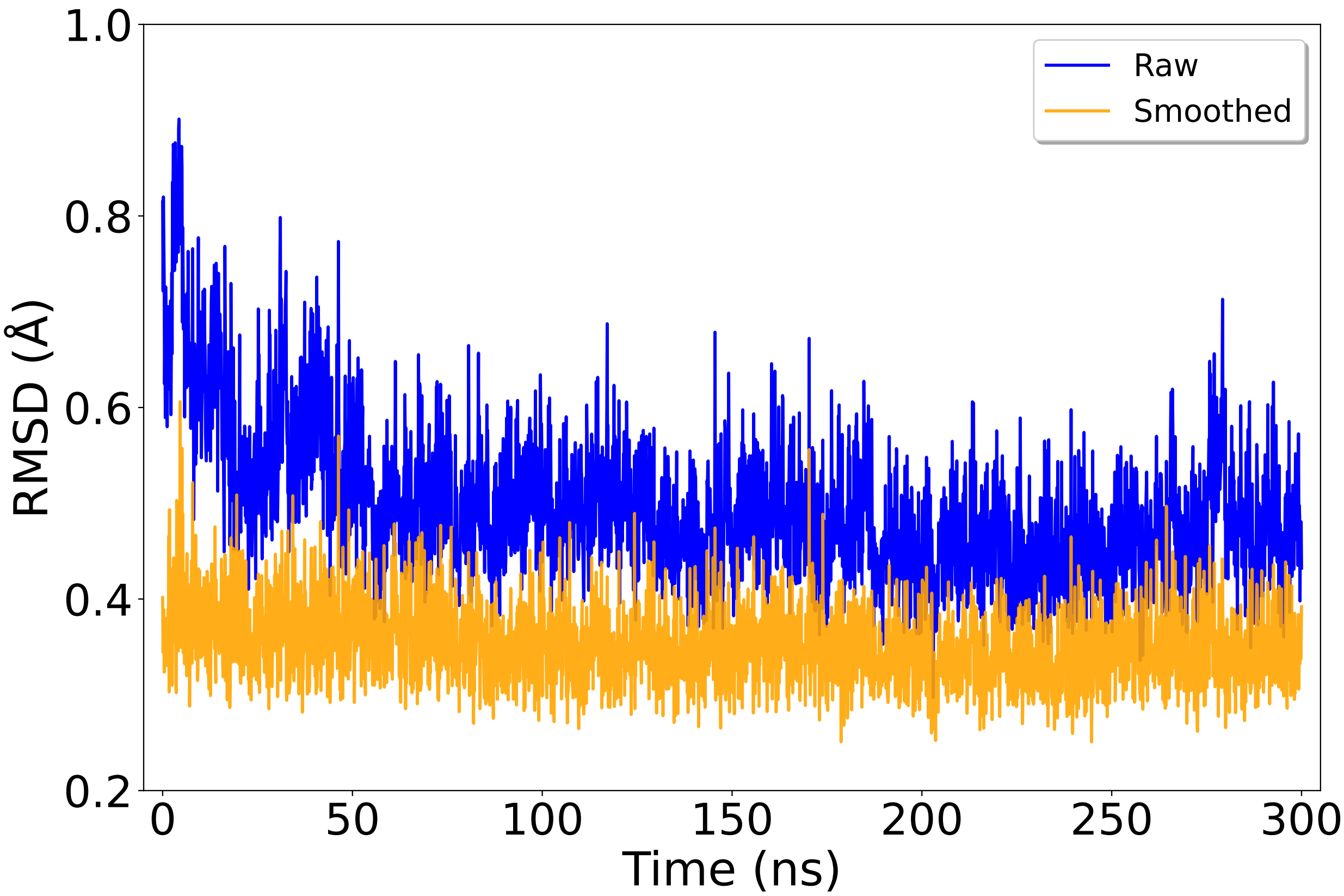

Figure S9: Time series of raw (blue) and smoothed (orange, W_{op} = 20 frames, 4 ns full triangular window) BRMSD for SND3 over 300 ns. Smoothing reduces the mean from 0.50 to 0.35 Å (30% reduction) and the standard deviation from 0.08 to 0.04 Å (50% reduction).

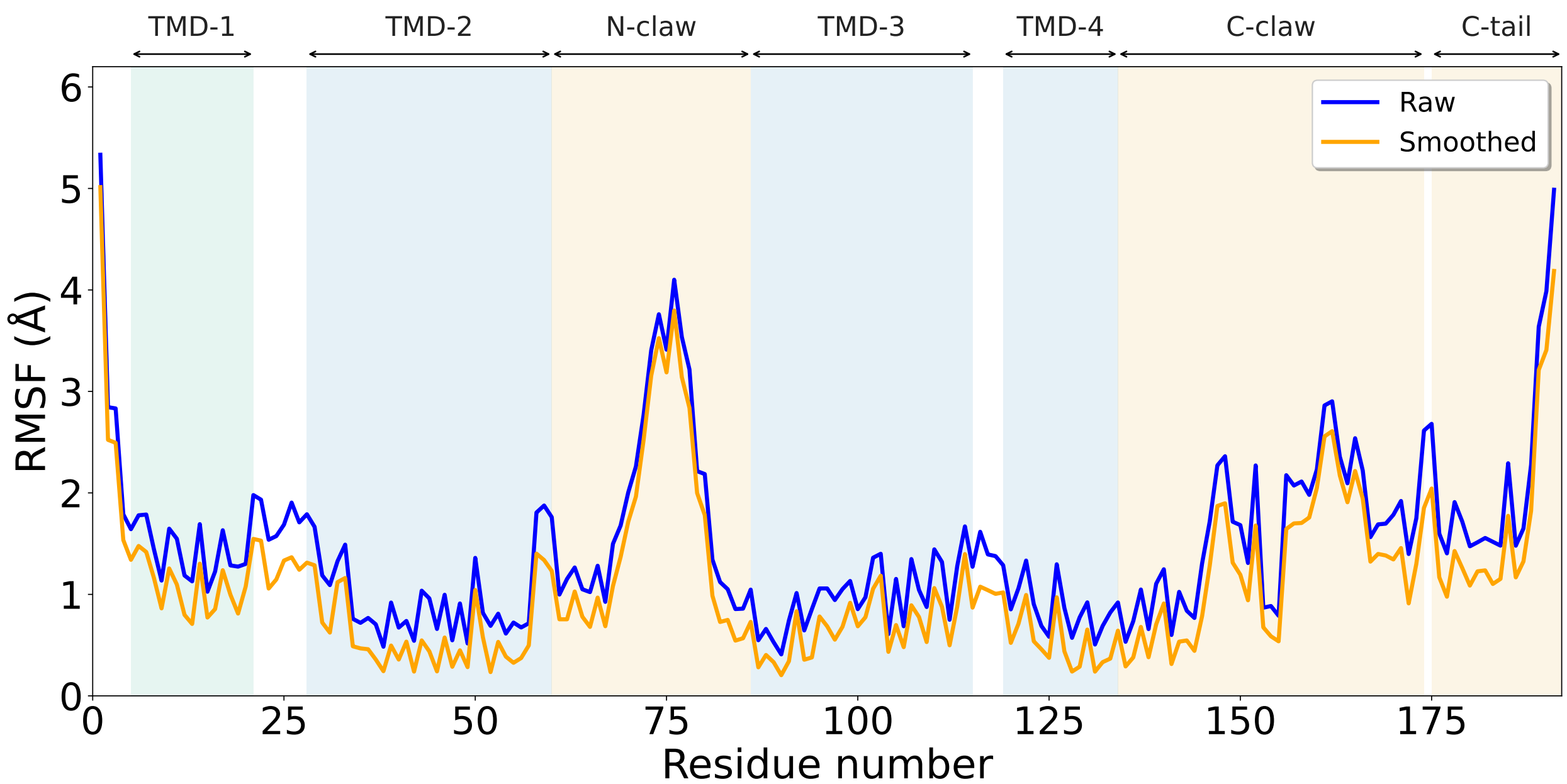

Figure S10: Per-residue RMSF for SND3 before (blue) and after (orange) smoothing at $W_{\text{op}} = 20$ frames. Smoothing reduces RMSF throughout the chain, and the largest absolute reductions occur in the mobile N-claw, C-claw, and C-tail regions. Domain shading follows the scheme in Fig. S3.

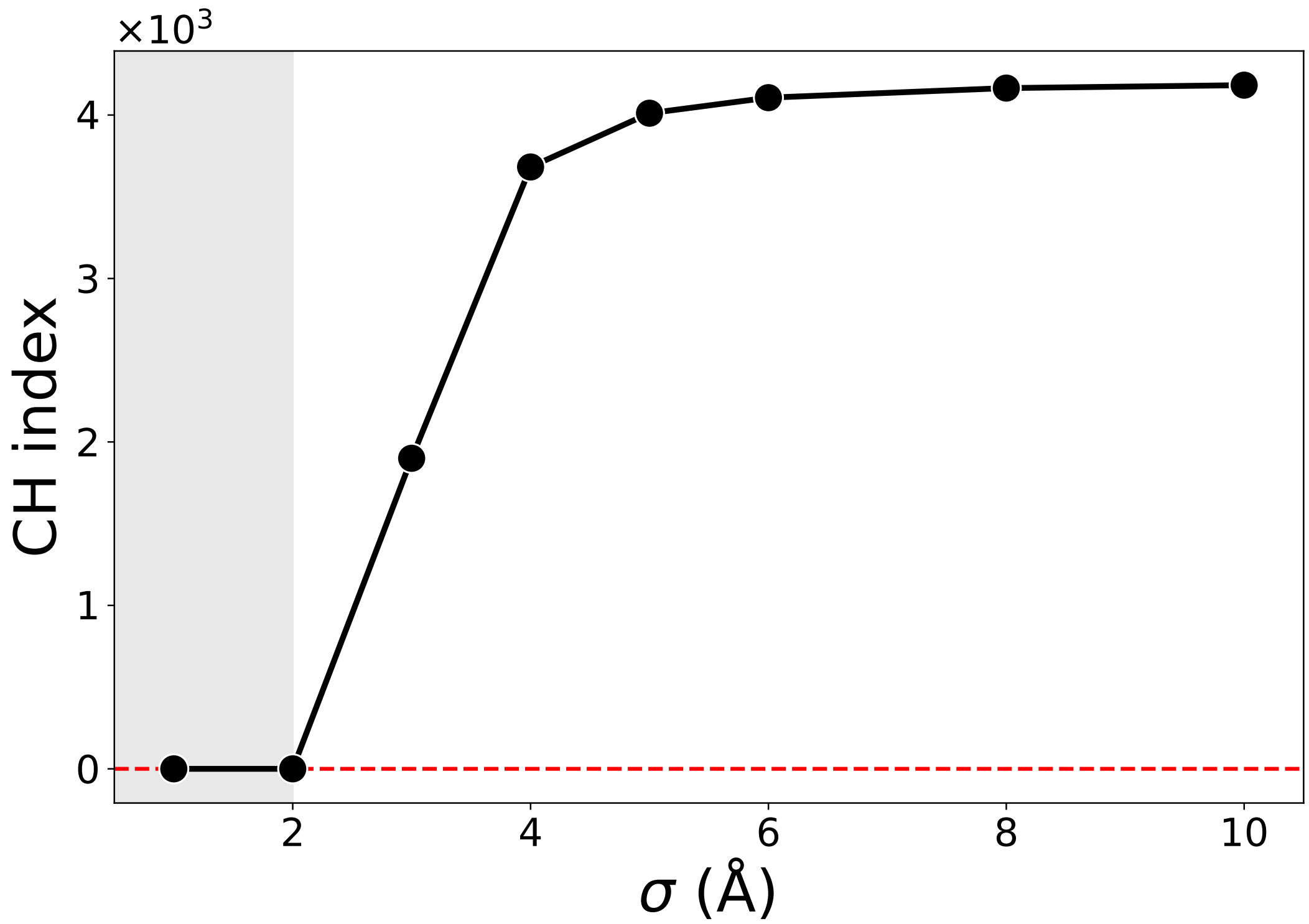

Figure S11: Clustering quality versus the alignment σ for the ADK DIMS ensemble ($K = 2$, $\tau = 5.0$ Å^2). The Calinski-Harabasz (CH) index collapses to zero for $\sigma \lesssim 2$ Å (shaded), where weight concentrates on the rigid CORE and the two cluster centres merge. CH index rises sharply at $\sigma = 3$–4 Å and plateaus for larger σ.

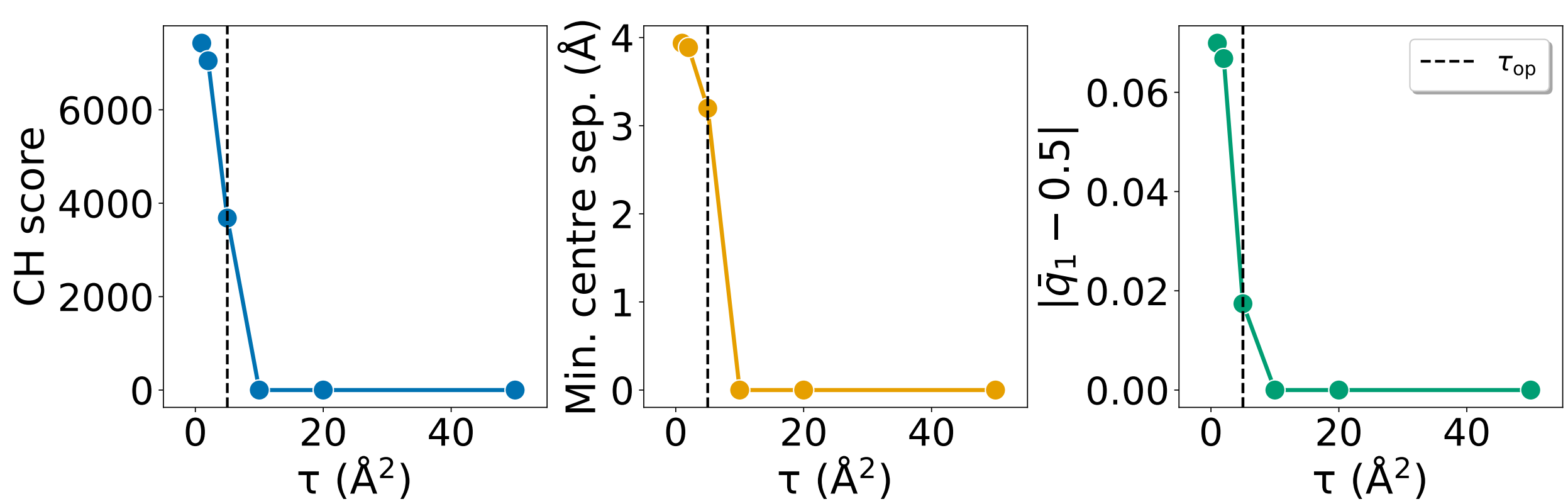

Figure S12: Results of the τ sweep at $K = 2$ for ADK DIMS clustering. Three panels show the Calinski-Harabasz (CH) index (left), the minimum cluster-centre separation in BRMSD units (centre), and the population imbalance $|\bar{q}_1 - 0.5|$ (right) versus τ. CH and centre separation stay high for $\tau \leq 5$ Å^2 and collapse to zero at $\tau \geq 10$ Å^2, where the responsibilities become uniform and the centres merge. The dashed line marks $\tau_{\text{op}} = 5.0$ Å^2, the largest τ that preserves discrimination and gives the most balanced partition.

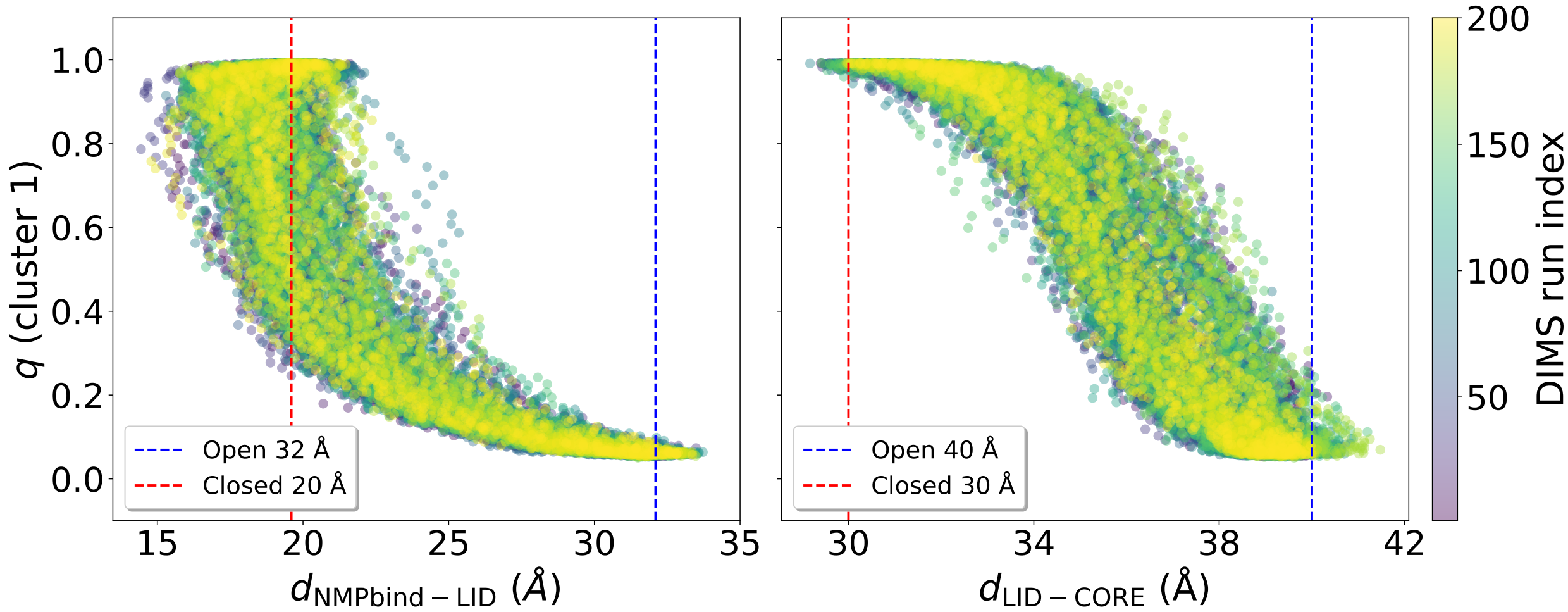

Figure S13: ADK cluster responsibilities $q(\text{cluster } 1|i)$ are plotted versus NMP-LID distance (left) and LID-CORE distance (right), colored by run index. Both order parameters correlate strongly with cluster membership (Pearson r = -0.88 and -0.94, respectively). Vertical lines mark the open (4AKE) and closed (1AKE) crystal-structure reference distances.

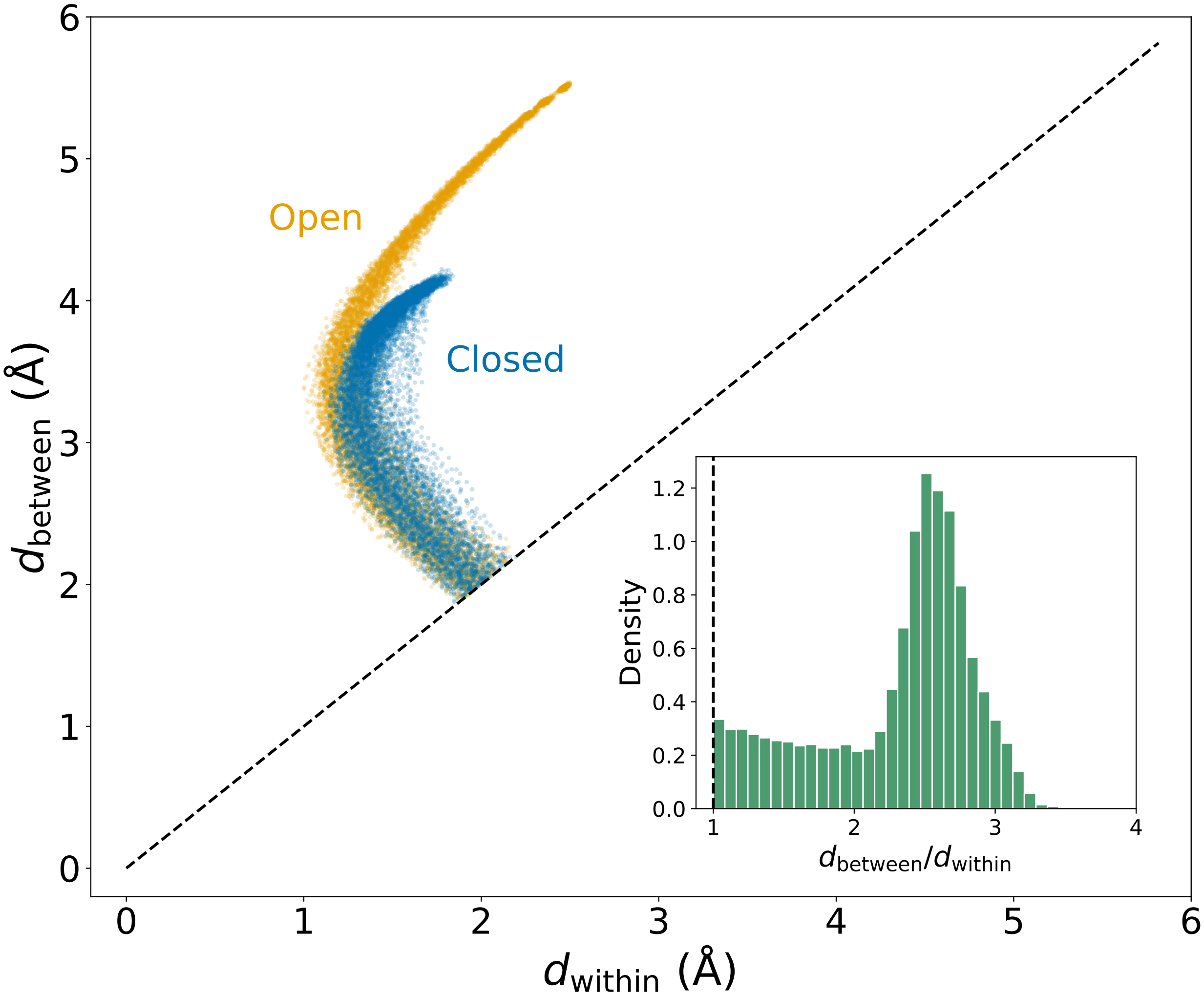

Figure S14: For ADK, within- and between-cluster BRMSD distances are compared in two panels. (Left) Scatter of d_{between} versus d_{within} colored by cluster assignment (orange: cluster 1, blue: cluster 2), where the dashed diagonal marks $d_{\mathrm{between}} = d_{\mathrm{within}}$. All frames satisfy $d_{\mathrm{between}} > d_{\mathrm{within}}$, confirming genuine cluster separation. Inset shows the probability density of the ratio $d_{\mathrm{between}}/d_{\mathrm{within}}$, with a median ratio of 2.48.

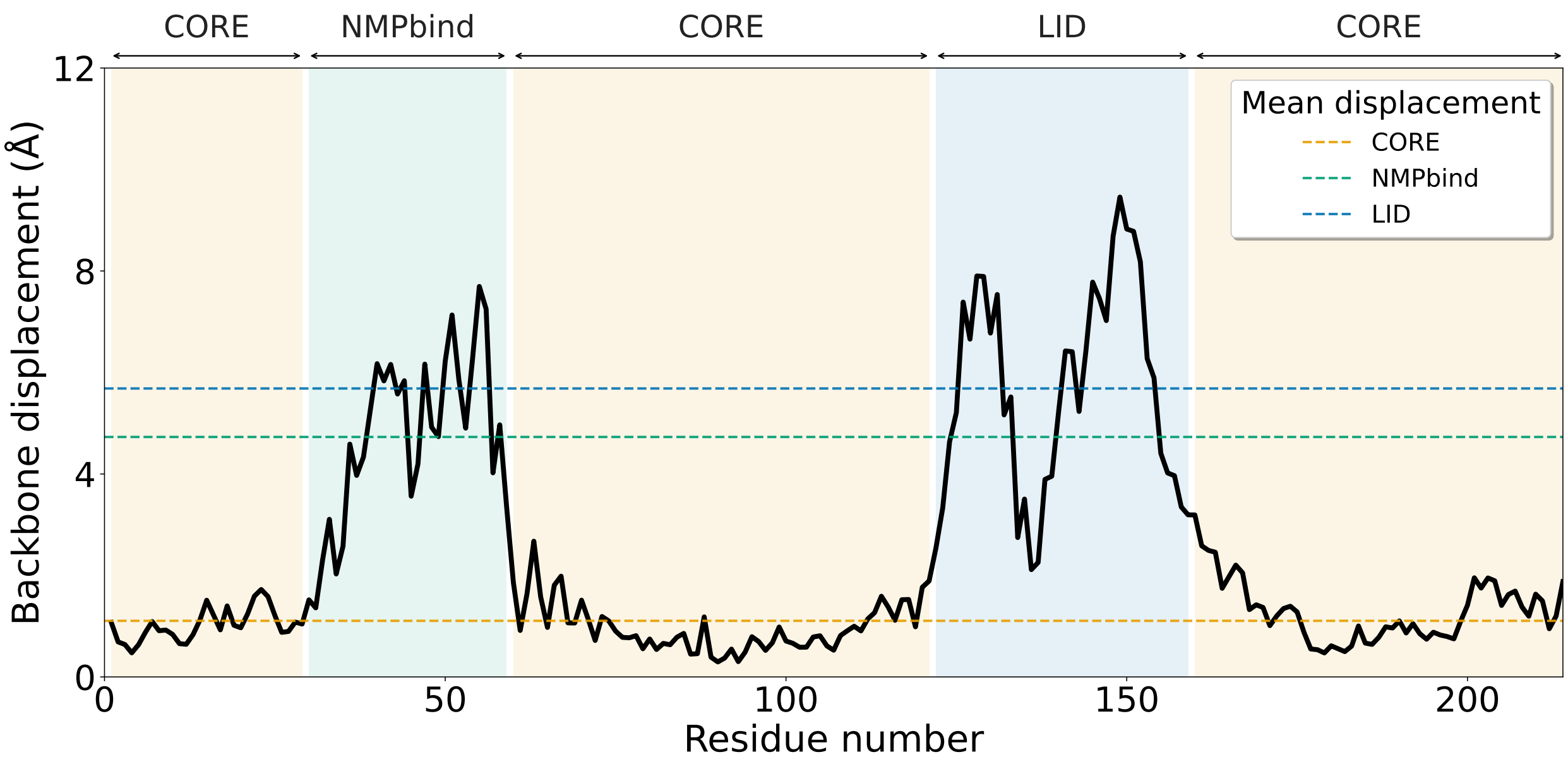

Figure S15: Per-residue displacement between the $K = 2$ cluster centres in ADK is shown as a function of residue number. Mean displacements of the CORE, NMPbind, and LID domains are 1.11 Å, 4.73 Å, and 5.68 Å, respectively. The hierarchy CORE < NMPbind < LID is consistent with the experimentally observed order of domain rigidity.

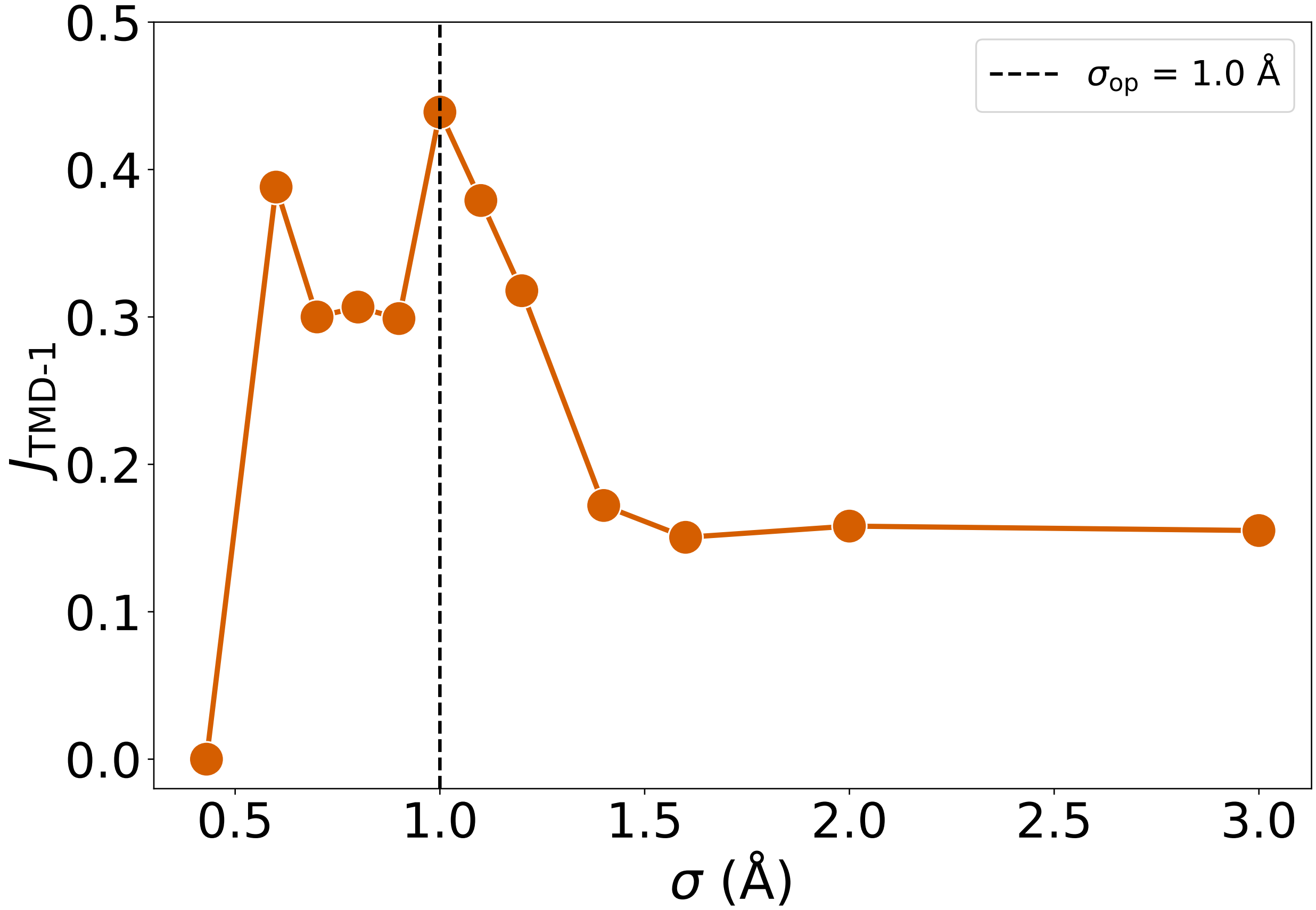

Figure S16: Jaccard index between the best-matching peeling round and the TMD-1 reference, $J_{\text{TMD-1}}$, as a function of the alignment σ for SND3 sequential peeling. The overlap peaks at $\sigma = 1.0$ Å (dashed line) and falls to a low plateau for $\sigma \geq 1.6$ Å, where TMD-1 merges with the groove.

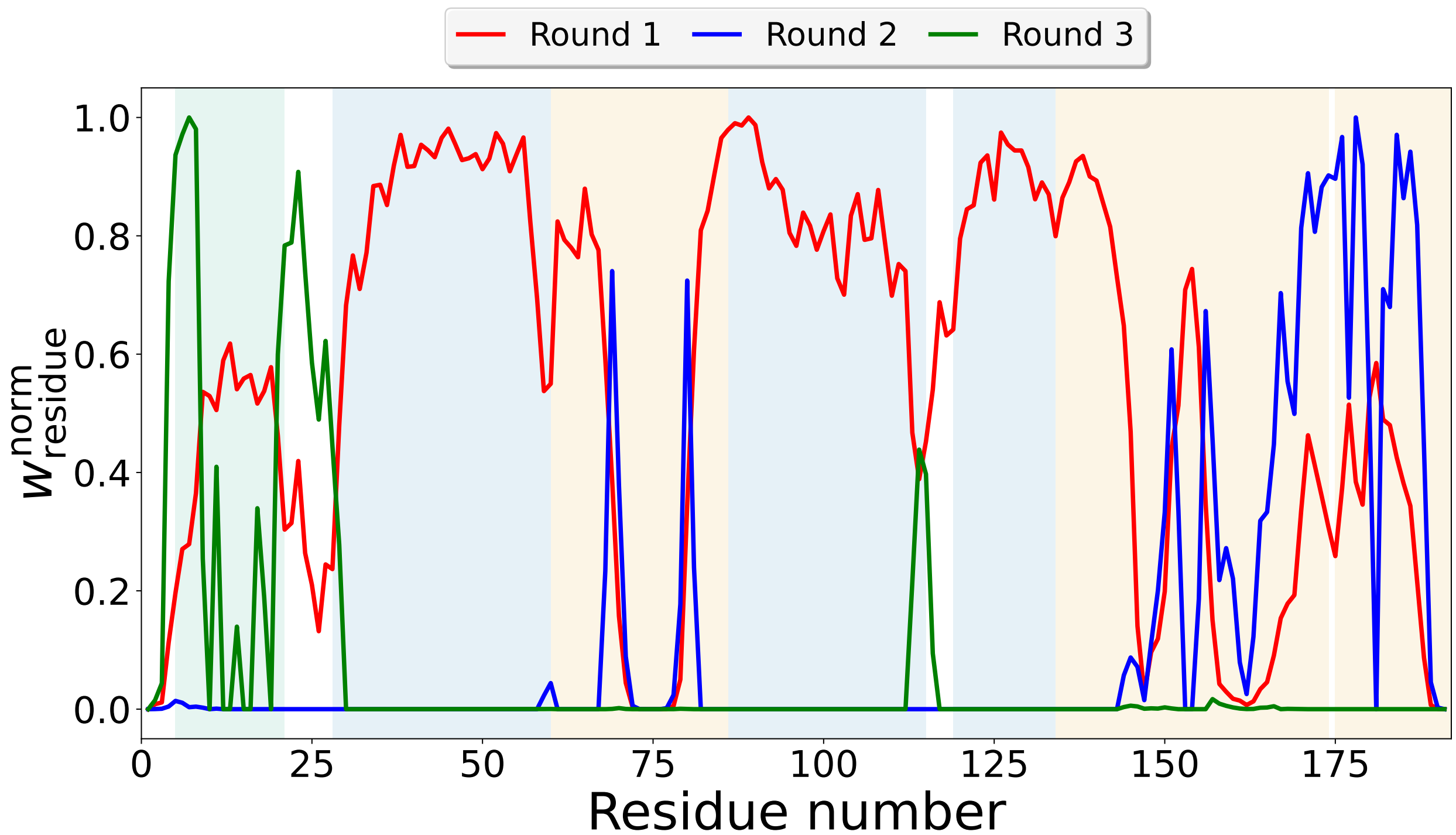

Figure S17: Per-residue weights $w_{\text{residue}}^{\text{norm}}$ (normalized within each round to its maximum) from SND3 sequential peeling at $\sigma = 1.0$ Å. Round 1 (red) concentrates on the TM groove, Round 2 (blue) on the cytosolic claws, and Round 3 (green) on TMD-1. Domain shading follows Fig. S3.

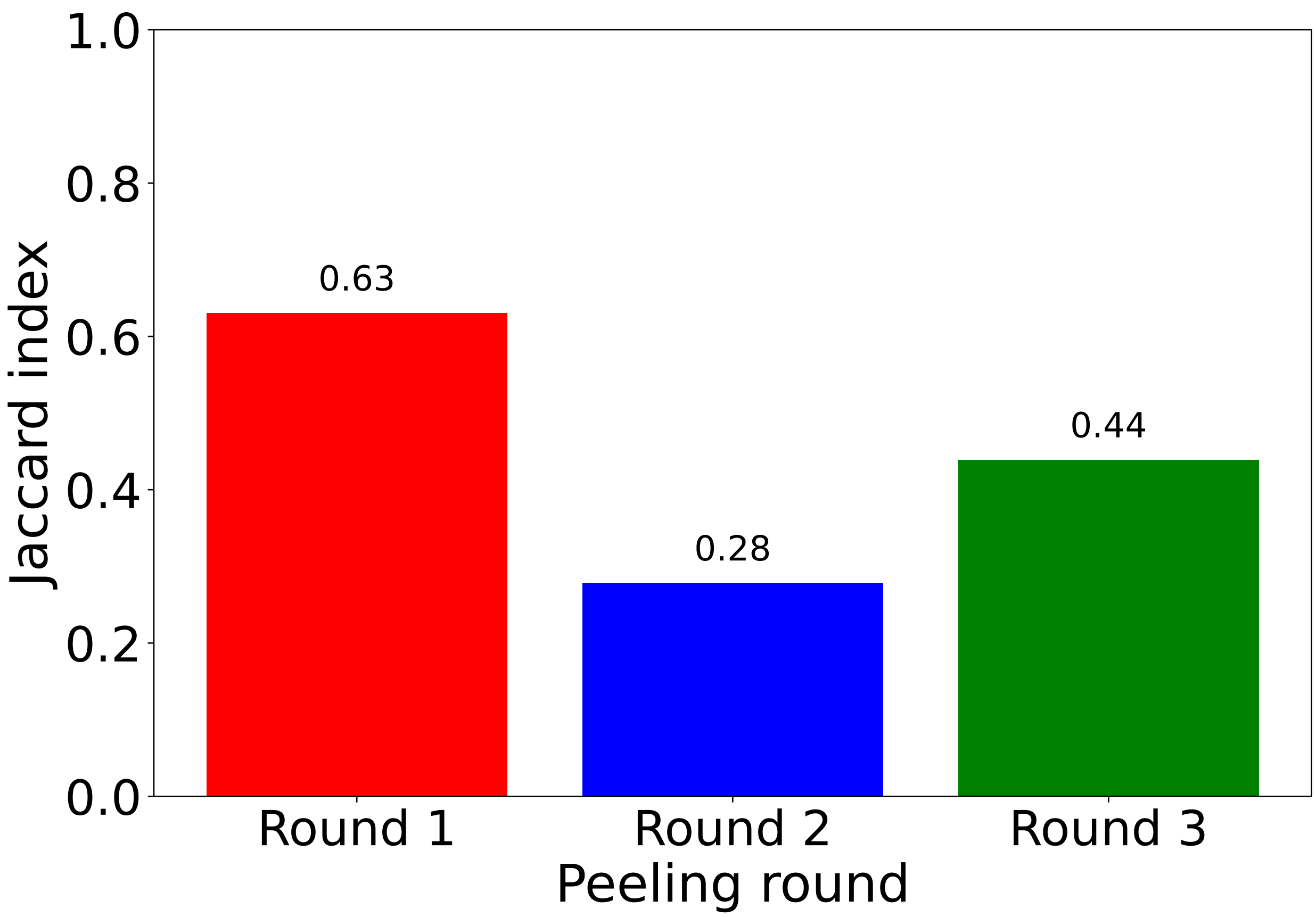

Figure S18: Jaccard index of each SND3 peeling round with its best-matching reference domain at $\sigma = 1.0$ Å. Round 1 recovers the TM groove and Round 3 recovers TMD-1, both distinct from the cytosolic Round 2.

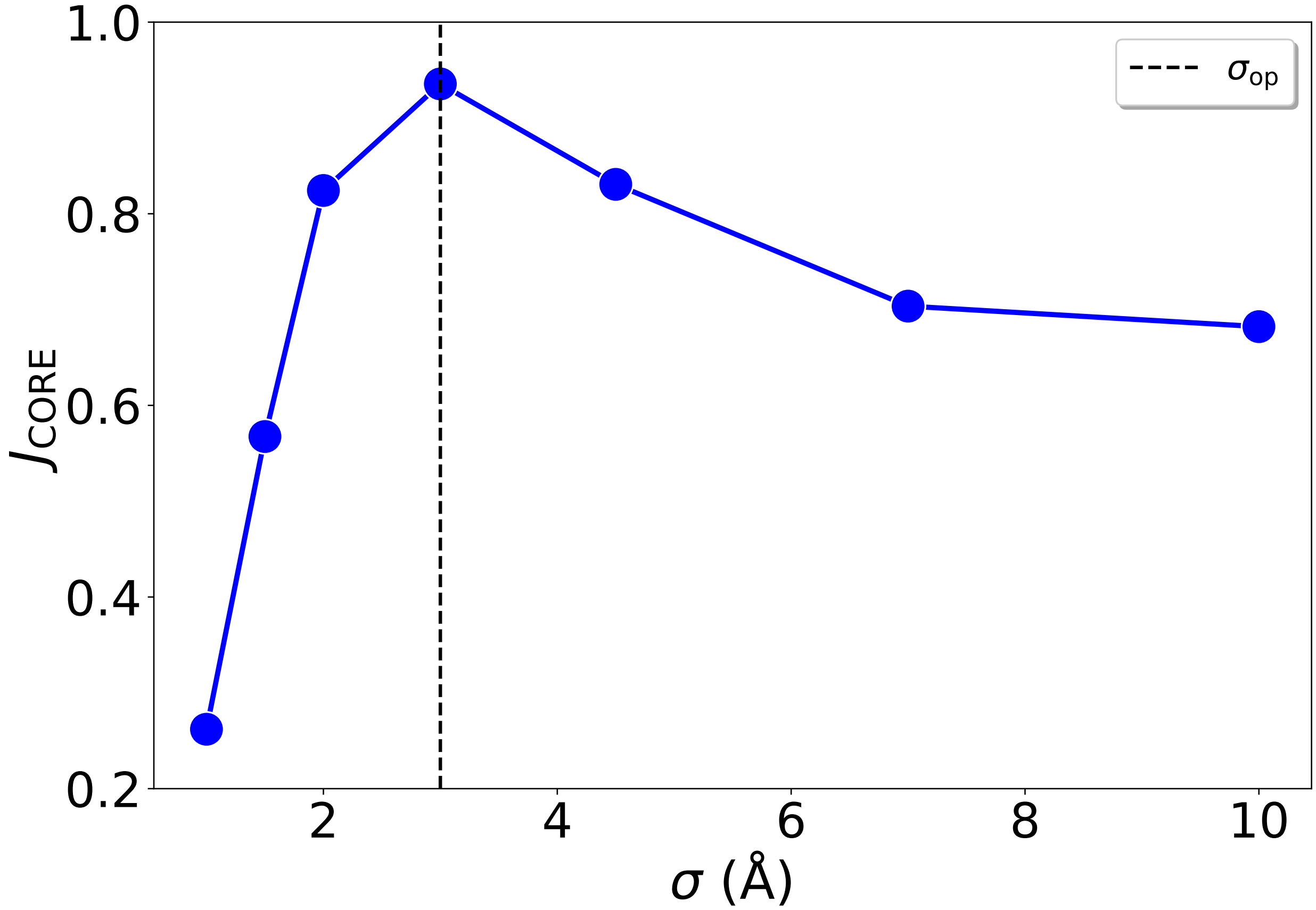

Figure S19: The Jaccard index of the recovered CORE domain, J_{CORE}, is plotted as a function of σ for the $K = 1$ sweep on the full 856-atom ADK FRODA pool. J_{CORE} peaks at $\sigma_{\text{op}} = 3.0$ Å (dashed line, $J_{\text{CORE}} = 0.94$) and decays at both larger and smaller σ, defining the operating point used for sequential peeling.

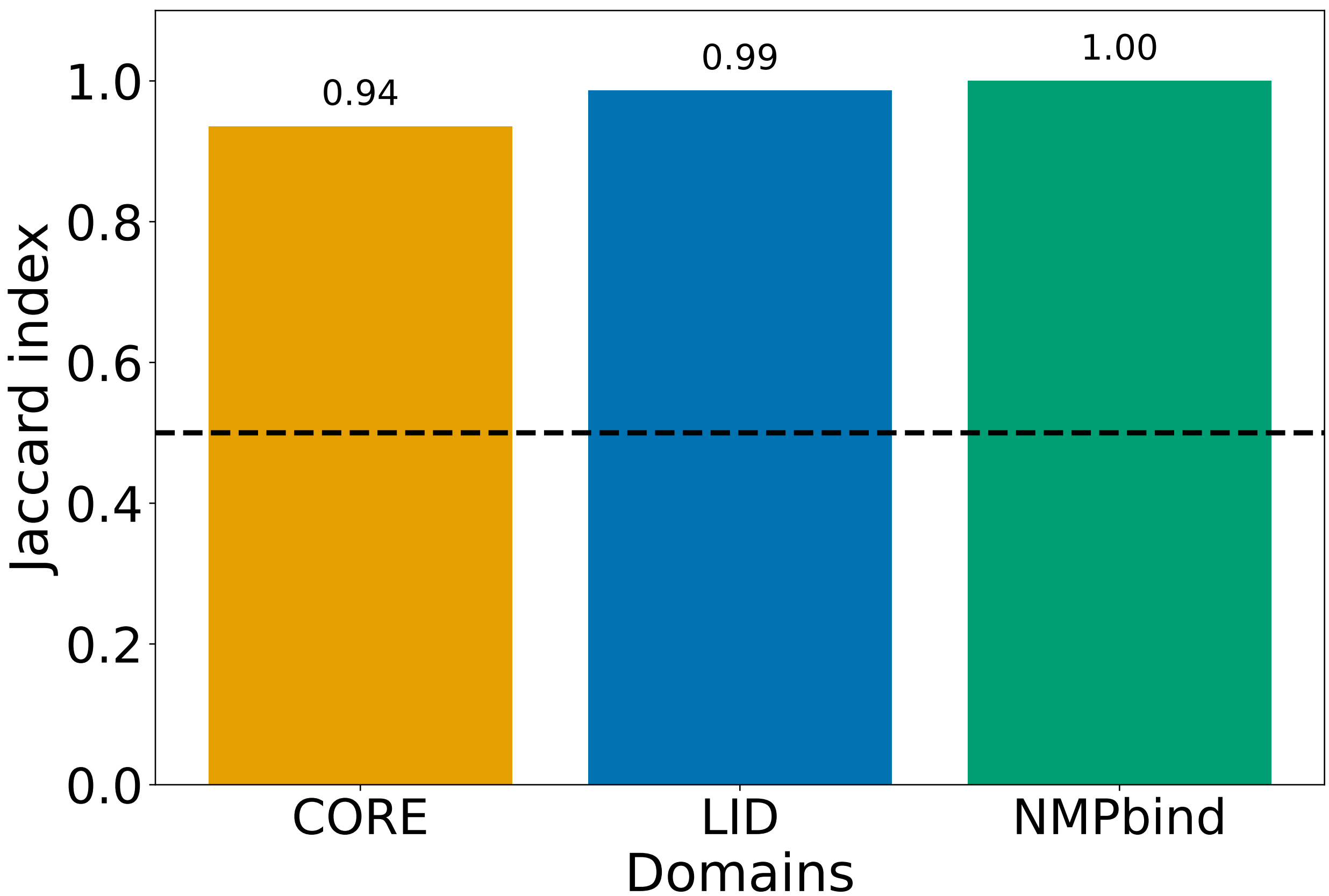

Figure S20: The Jaccard index for each of the three sequential-peeling steps of rigid domain detection for ADK is shown, based on 28,282 FRODA backbone frames ($\sigma_{\mathrm{op}} = 3.0$ Å, $N = 856$). The recovered Jaccard indices are $J_{\mathrm{CORE}} = 0.94$ (Step 1, 856 atoms), $J_{\mathrm{LID}} = 0.99$ (Step 2, 240 non-CORE atoms), and $J_{\mathrm{NMPbind}} = 1.00$ (Step 3, 96 remaining atoms). The dashed line marks the $J = 0.5$ recovery threshold.

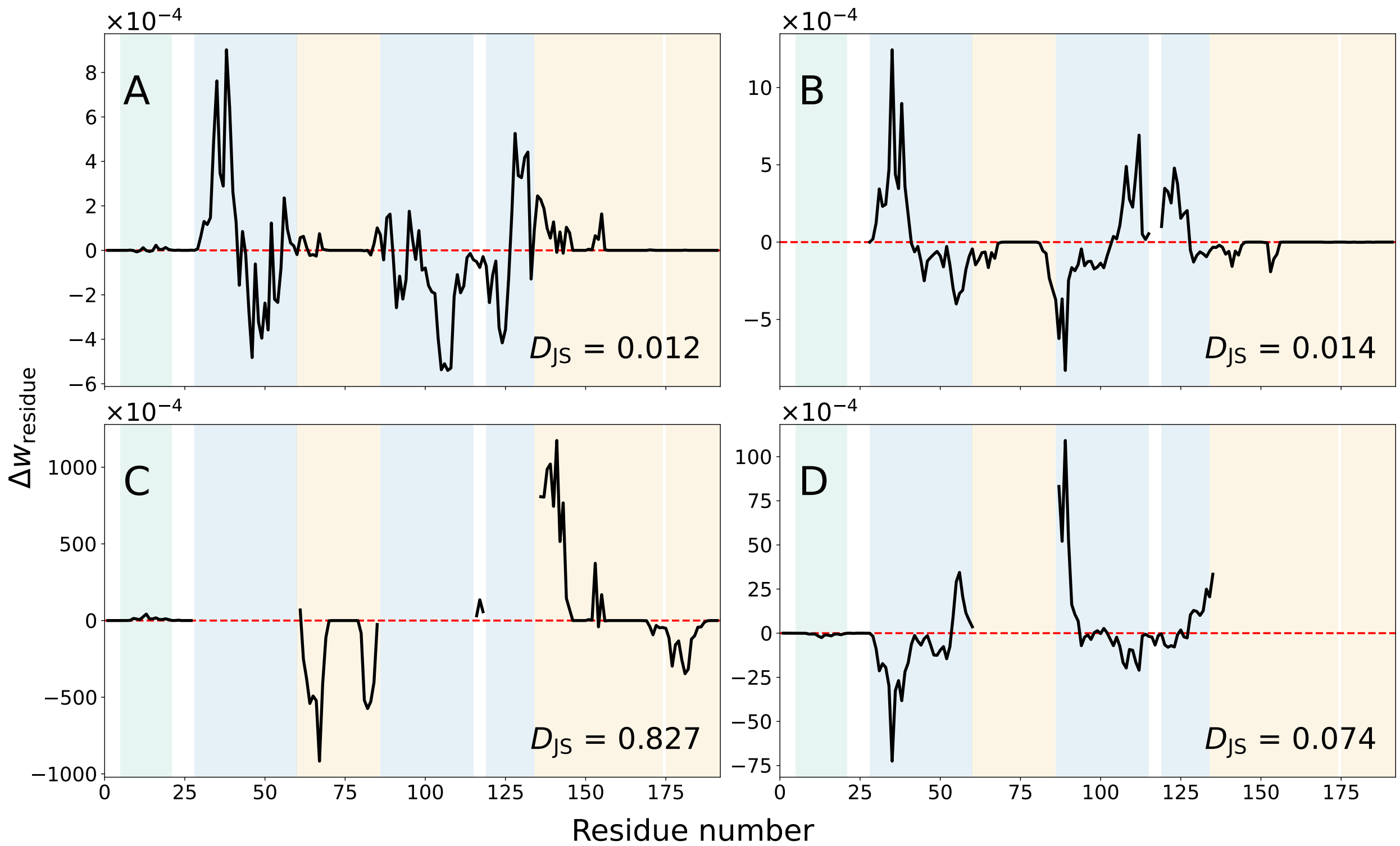

Figure S21: Consistency of the SND3 alignment weights at $\sigma_{op} = 0.43$ Å. Each panel shows the per-residue weight difference $\Delta w_{residue}$ between two optimizations. (A) M-invariance: all 3001 frames versus every other frame (1501 frames), $D_{JS} = 0.014$. (B)-(D) Pool-invariance: a whole region is removed from the selection and the weights are re-optimized on the retained atoms, compared to the corresponding atoms of the full solution. Removing TMD-1 (B, $D_{JS} = 0.014$) or the cytosolic claws (D, $D_{JS} = 0.076$) leaves the retained weights unchanged, whereas removing the rigid TM groove (C, $D_{JS} = 0.836$) forces a different solution. Domain shading follows Fig. S3.

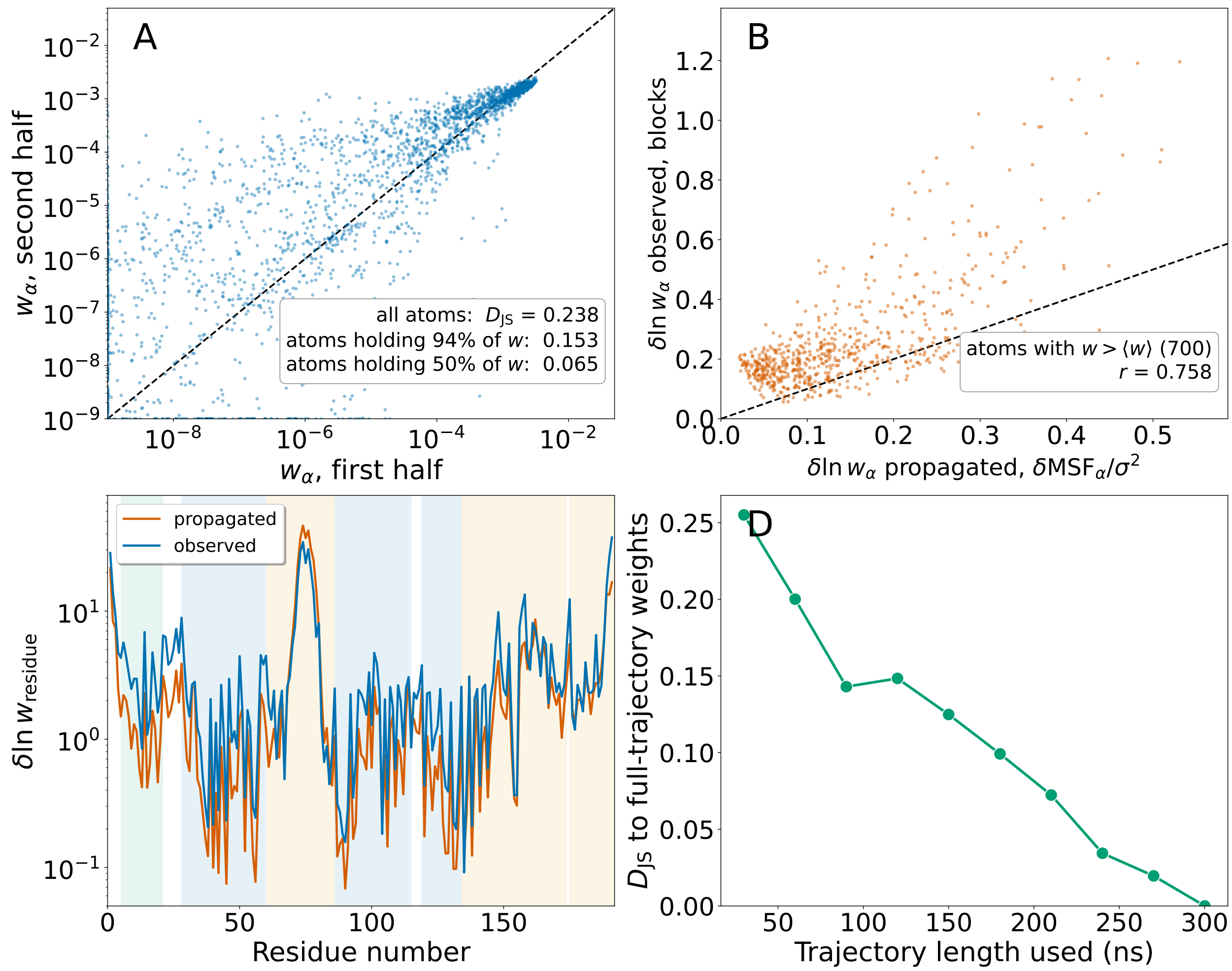

Figure S22: Finite-sampling uncertainty of the SND3 weights at $\sigma_{op} = 0.43$ Å. (A) Per-atom weights optimized independently on the first and second halves of the trajectory, with the Jensen-Shannon distance over all atoms and over the subsets carrying 94% and 50% of the total weight. The scatter is confined to the low-weight tail; the atoms that carry the alignment lie near the diagonal. (B) Per-atom log-weight uncertainty propagated from the block standard error of the MSF through eq 27, against the spread observed when the weights are re-optimized on each of ten blocks, for the 700 atoms with above-average weight. The propagated estimate is first order at a fixed reference and so is systematically the smaller of the two. (C) Per-residue log-weight uncertainty on a logarithmic scale, propagated (orange) and observed (blue). Domain shading follows Fig. S3. (D) Jensen-Shannon distance between the weights from the first f of the trajectory and the full-trajectory weights, against the length used.

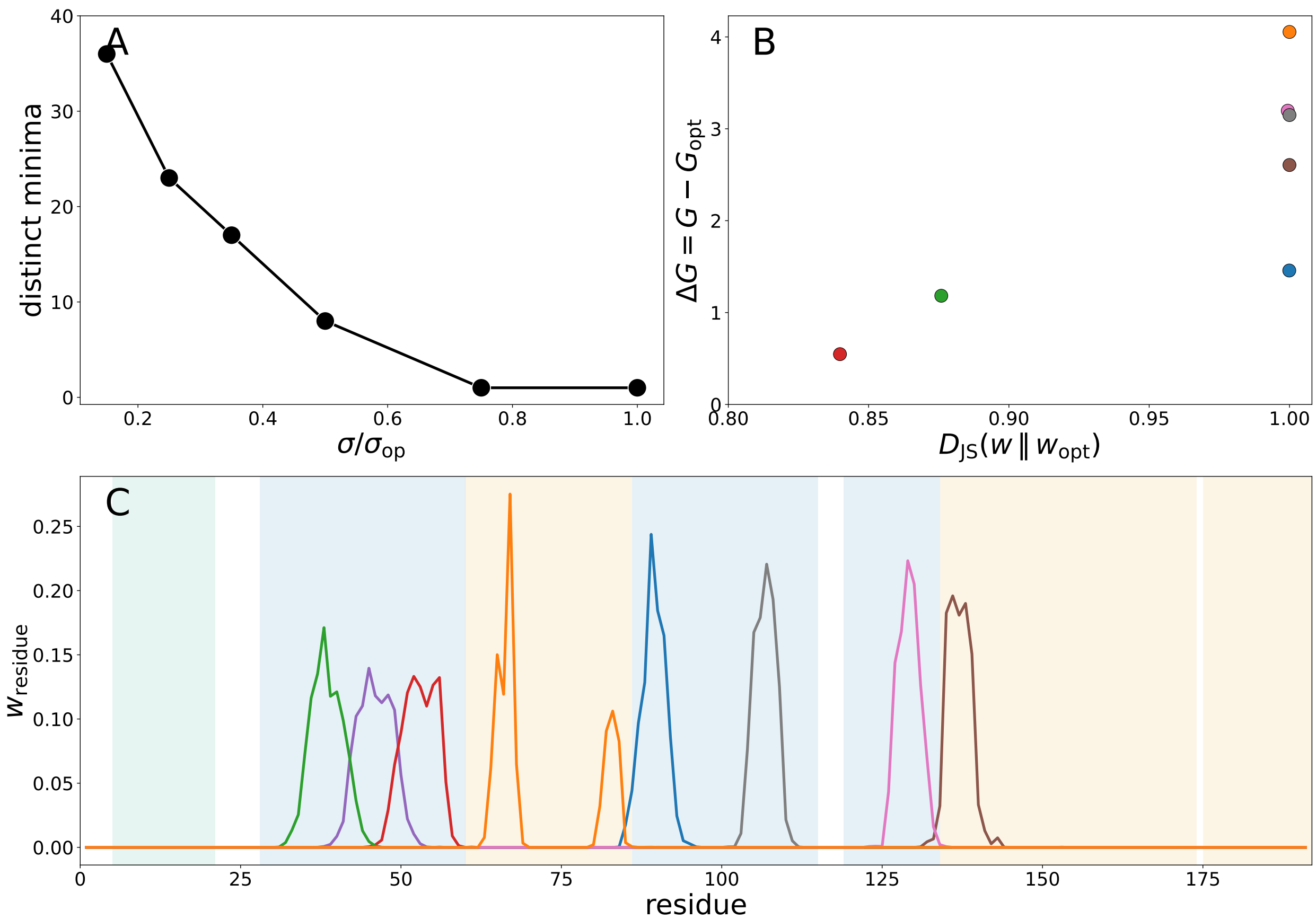

Figure S23: Fixed-point weight solutions of the SND3 alignment ($\theta = M\sigma^2$), obtained from 40 localized (Gaussian-bump) initializations clustered by Jensen-Shannon distance. (A) Number of distinct minima as a function of $\sigma/\sigma_{\text{op}}$. At σ_{op}, there is a single solution. It fragments into many as σ decreases. (B) Free-energy offset $\Delta G = G - G_{\text{opt}}$ versus $D_{\text{JS}}(\boldsymbol{w} \,\|\, \boldsymbol{w}_{\text{opt}})$ at $0.5\,\sigma_{\text{op}}$, showing the 7 higher-G alternative minima. The global solution ($\Delta G = 0$) is not shown. All alternatives have higher G than the global minimum. (C) Per-residue weight profiles of the 8 minima at $0.5\,\sigma_{\text{op}}$, each localized on a different rigid sub-structure.

Supplementary Movies

Movie S1. Raw (left) and BRMSD-aligned (right) SND3 trajectories are shown side by side ($\sigma_{\text{op}} = 0.43$ Å). The lighter colors represent the weight-concentrated transmembrane groove.

Movie S2. Raw (left) and smoothed (right) SND3 trajectories are shown side by side ($W_{\text{op}} = 20$ frames, 4 ns full triangular window, $\sigma_{\text{op}} = 0.43$ Å). Sub-nanosecond fluctuations are suppressed while slow movements are preserved.

References

(S1) Caliński, T.; Harabasz, J. A dendrite method for cluster analysis. *Commun. Stat.* **1974**, *3*, 1–27.

(S2) Michaud-Agrawal, N.; Denning, E. J.; Woolf, T. B.; Beckstein, O. MDAnalysis: A toolkit for the analysis of molecular dynamics simulations. *J. Comput. Chem.* **2011**, *32*, 2319–2327.

(S3) Gowers, R. J.; Linke, M.; Barnoud, J.; Reddy, T. J. E.; Melo, M. N.; Seyler, S. L.; Domański, J.; Dotson, D. L.; Buchoux, S.; Kenney, I. M.; Beckstein, O. MDAnalysis: A Python package for the rapid analysis of molecular dynamics simulations. Proceedings of the 15th Python in Science Conference. 2016; pp 98–105.
